# Axillary De-Escalation After Neoadjuvant Therapy in cN2/N3 HER2-Positive and Triple-Negative Breast Cancer: Navigating Biological Promise and Clinical Uncertainty

**DOI:** 10.3390/cancers18182986

**Published:** 2026-09-15

**Authors:** Kefah Mokbel, Ibrahim Abugheida, Janhavi Venkataraman, Humaid O. Al-Shamsi

**Affiliations:** 1 London Breast Institute, The Princess Grace Hospital, London W1U 5NY, UK; kefahmokbel2@gmail.com; 2 Burjeel Cancer Institute, Abu Dhabi P.O. Box 92510, United Arab Emirateshumaid.al-shamsi@medportal.ca (H.O.A.-S.); 3 Dana-Farber Cancer Institute and Harvard Medical School, Boston, MA 02215, USA

**Keywords:** breast cancer, axillary lymph node dissection, neoadjuvant chemotherapy, sentinel lymph node biopsy, regional nodal irradiation

## Abstract

Axillary de-escalation after neoadjuvant systemic therapy (NST) is established in cN1 breast cancer, but the trials validating sentinel lymph node biopsy (SLNB), targeted axillary dissection (TAD) and omission of regional nodal irradiation (RNI) largely excluded cN2/N3 disease. In HER2-positive and triple-negative disease, NST achieves axillary pathological complete response (pCR) in about one-third of cN2/N3 patients and 70–80% of those achieving breast pCR—establishing biological plausibility, not technical or trial-validated eligibility. The prospective evidence base comprises 87 patients evaluated against completion axillary lymph node dissection (cALND), only 41 informative for false-negative rate. Much of this evidence is defined by node count rather than by matting or fixation, so it applies directly to patients with more than three suspicious but mobile nodes—AJCC cN1 by fixation criteria, but sharing the same multi-node technical problem—while being correspondingly less confined to AJCC-defined cN2/N3 than the label implies. Across non-randomised cohorts, no oncological penalty has emerged after de-escalation in selected responders, though only three supply an axillary-recurrence numerator confined to advanced nodal disease. Within these limits, omission of cALND may reasonably be offered to selected excellent responders with cN2a or level I/II-predominant disease and TAD-confirmed nodal pCR, provided RNI is retained, and the decision is multidisciplinary-team supported and documented. Omitting RNI as well rests on extrapolation from NSABP B-51/RTOG 1304, which excluded this population, and should remain within prospective evaluation, as should cALND omission for residual disease. Level I/II TAD/SLNB does not sample the compartments defining cN2b, cN3a, or cN3c disease and samples only the axillary component of cN3b disease, which cALND does not sample either; comprehensive RNI remains indicated in these subgroups. In cN2b, the axilla is clinically uninvolved, so cALND is not required, and axillary staging by SLNB alone is appropriate. cN2/N3-specific validation of false-negative rate and outcomes remains the priority.

## Simple Summary

Some breast cancers have already spread extensively to the lymph nodes under the arm when they are diagnosed. Modern drug treatment given before surgery can clear the cancer from these nodes completely, most often in triple-negative and HER2-positive breast cancer. The standard operation for such patients removes all lymph nodes, which can cause lasting problems including arm swelling and shoulder stiffness. We reviewed whether a smaller operation, sampling only a few nodes, is enough. No study has yet shown worse outcomes when the smaller operation is used in patients who respond very well, but its accuracy has not been properly tested in this group. Our reading of the evidence is that, for carefully chosen patients whose nodes appear clear on scans and are then confirmed free of cancer by the smaller operation, avoiding full clearance is a reasonable option for discussion, provided radiotherapy to the area is still given and the decision is made jointly by the patient and the whole treatment team. Leaving out radiotherapy as well is a further step that should for now be taken only within clinical trials.

## 1. Introduction

The American Joint Committee on Cancer (AJCC) stages clinically node-positive breast cancer as cN1 (mobile level I/II axillary nodes), cN2 (matted/fixed level I/II nodes, or clinically apparent internal mammary nodes without axillary involvement), or cN3 (infraclavicular, supraclavicular, or internal mammary involvement with axillary disease) [1]. AJCC further subdivides cN2 into cN2a (fixed/matted level I/II axillary nodes) and cN2b (internal mammary nodes without axillary involvement) and cN3 into cN3a (infraclavicular), cN3b (internal mammary plus axillary), and cN3c (supraclavicular) [1]. These clinical categories are defined by nodal fixation and anatomic compartment on examination and imaging, not by a specific node count, and matting/fixation is inherently subject to interobserver variability. For the purposes of this review, we additionally incorporate a pragmatic, count-based imaging definition used by several of the cohorts discussed below—most notably the MARI protocol—in which four or more suspicious nodes on fluorodeoxyglucose positron emission tomography/computed tomography (FDG-PET/CT) are treated as a surrogate for extensive (cN2/N3-equivalent) nodal disease, independent of formal AJCC matting/fixation criteria. Because a PET-defined four-or-more-node cohort and an AJCC-defined matted/fixed cN2 cohort are not necessarily biologically or technically equivalent populations, this distinction is made explicit wherever the underlying studies rely on it, rather than assuming a single uniform cN2/N3 definition across the evidence base.

cN2/N3 should not, in any case, be regarded as a homogeneous axillary category. Only cN2a—matted/fixed level I/II disease—falls within the compartment that level I/II targeted axillary dissection (TAD) or sentinel lymph node biopsy (SLNB) actually samples; cN2b (internal mammary only), cN3a (infraclavicular), and cN3c (supraclavicular) lie outside it entirely, and cN3b, which combines internal mammary with level I/II axillary involvement, is only partly addressed by a level I/II procedure, since its internal mammary component remains unsampled regardless of axillary response. Emerging evidence that post-neoadjuvant chemotherapy (NAC) imaging response of visible internal mammary nodes on MRI may itself act as a surrogate for internal mammary chain (IMC) involvement offers a potential non-invasive route to addressing this gap, though this remains investigational and requires prospective validation before it could inform clinical practice [2]. The feasibility and oncological implications of de-escalation discussed throughout this review therefore differ substantially by AJCC subcategory, and most of the direct evidence available applies to cN2a and other level I/II–predominant presentations rather than to the full cN2/N3 spectrum—a caveat that applies throughout and is revisited in the Section 9. A high pathological response rate remains, in any subcategory, a necessary but not sufficient condition for surgical de-escalation—a distinction that structures this review.

Axillary surgery has progressively de-escalated over the past two decades, from routine complete axillary lymph node dissection (cALND) to SLNB in clinically node-negative disease, and to TAD/SLNB after neoadjuvant systemic therapy (NST) in selected cN1 patients, reflecting its primarily staging role and its dose-related morbidity (lymphoedema, restricted shoulder motion and numbness) [3].

The trials underpinning this de-escalation paradigm in patients receiving NST (ACOSOG Z1071 [4], SENTINA [5], SN-FNAC [6], GANEA2 [7], the TAD registry [8], Alliance A011202 [9], NSABP B-51/RTOG 1304 [10]) were designed around cT1–3N1 disease; cN2/N3 patients were excluded or represented a marginal fraction of enrolment. Current NCCN [11] and ESMO [12] guidelines therefore retain cALND (±RNI) as standard for cN2a and axillary cN3 disease irrespective of NST response—a recommendation that predates modern dual-targeted anti-HER2 and checkpoint-inhibitor regimens, under which breast and axillary pCR rates in HER2-positive breast cancer and triple-negative breast cancer (TNBC) now frequently exceed 50%. A recent cN2/N3-specific NCDB series reports ypN0 rates of 70% for HER2-positive tumours and 57% for TNBC [13], raising the question of whether a substantial proportion of these patients may be candidates for axillary treatment de-escalation, though appropriate selection criteria remain unvalidated.

This review is restricted to HER2-positive disease and TNBC, the most NST-sensitive and therefore most plausible subtypes for de-escalation; hormone-receptor-positive/HER2-negative tumours achieve substantially lower pathological complete response (pCR) rates and are not addressed further. Where mixed-subtype cohorts are cited, HER2-positive- and TNBC-specific findings are reported where available.

**Aims of This Review.** We address three linked questions for cN2/N3, HER2-positive breast cancer or TNBC treated with NST: (i) can cALND be replaced by TAD/SLNB after an excellent radiological/pathological response; (ii) if nodal pCR is achieved, can RNI alone substitute for axillary surgery, and can the B-51/RTOG 1304 finding regarding RNI omission in cN1→ypN0 disease be extrapolated to cN2/N3; (iii) with residual axillary disease, is RNI including the undissected axilla an appropriate substitute for cALND, pending the results of Alliance A011202 and TAXIS [14,15], neither of which fully answers this question for cN2/N3?

We separate two questions often conflated in this literature: whether cN2/N3 tumours respond well to NST biologically and whether responders can be identified accurately enough to safely reduce axillary surgery—with technical, immunological, and trial-evidence questions addressed in turn below. Table 1 summarises why cN1 de-escalation trials cannot be directly extrapolated to cN2/N3; the full evidence base is summarised in Table 2.

## 2. Methods (Search Strategy)

This was an expert-led narrative review following the SANRA framework [33], not a systematic review; no protocol was registered, and the search below, while structured, does not follow PRISMA methodology or report a formal screening flow. PubMed/MEDLINE and Google Scholar were searched through 31 August 2026 using combinations of “clinical N2,” “clinical N3,” “axillary pathologic complete response,” “targeted axillary dissection,” “sentinel lymph node biopsy,” “neoadjuvant,” “regional nodal irradiation,” “HER2-positive,” and “triple-negative breast cancer.” Reference lists, major consensus statements (St Gallen 2019/2021 [34]), and ClinicalTrials.gov were hand-searched for ongoing/unpublished trials. The initial literature search conducted using PubMed and Google Scholar identified a total of 455 records after removal of duplicates. Titles and abstracts were screened by the authors against the prioritisation criteria set out below, and relevance was determined by consensus rather than by formal eligibility criteria applied in duplicate; 57 articles were retained. These counts describe the search narratively and are not offered as a PRISMA screening flow.

**Study Selection.** Articles reporting nodal pathological response, FNR, or oncological outcomes after NST, in English, were prioritised based on the following: (1) cN2/N3-specific reporting; (2) prospective design where available, though retrospective cohorts were included given the paucity of prospective cN2/N3 data; (3) oncological or FNR endpoints over purely descriptive series; and (4) use of contemporary HER2-directed or immunotherapy-containing regimens where reported. Where multiple analyses of the same dataset existed (e.g., successive TAXIS or A011202 substudies), only the most complete report was retained for detailed synthesis. Conference abstracts were included only for major cooperative-group trials lacking a peer-reviewed manuscript, and are identified as such in the reference list. cN0/cN1-only studies were retained only where required for context or where their exclusion of cN2/N3 is itself central to the argument. No formal risk-of-bias tool was applied, given the narrative format; this limitation is noted below. The completed SANRA self-assessment for this review is provided in Table S1 of the Supplementary Materials.

## 3. Nodal Pathological Response in cN2/N3 Disease Under Contemporary Systemic Therapy

Six cN2/N3-specific cohorts anchor this review. A retrospective cN2 series restricted to patients with matted nodes or ≥3 suspicious nodes on ultrasound (*n* = 221) [16] reported axillary pCR rates of 67.9% in HER2-positive disease, 45.2% in TNBC, and 16.8% in hormone receptor (HR)-positive/HER2-negative tumours, with subtype being the strongest predictor of nodal response. A larger multicentre cN2 cohort restricted to HER2-positive disease and TNBC (CSBrS-012, *n* = 709) [17] reported a 25% breast pCR rate and a 36.4% axillary pCR rate overall, rising to 78.0% among patients achieving breast pCR: 83.8% in HR-negative/HER2-positive disease, 75.0% in triple-positive (HR-positive/HER2-positive) disease—both variants falling within this review’s HER2-positive scope—and 73.7% in TNBC (Table 3); the relative risk of residual nodal disease was 12.4 (95% CI 8.1–19.1) without breast pCR, suggesting that breast pCR is a robust predictor of axillary pCR. An NCDB analysis of cN2/N3 disease (*n* = 20,402) [13] found an overall ypN0 rate of 36.9% (70% in HER2+, 57% in TNBC and 29% in HR+/HER2-), yet 60.8% of patients—including many with nodal pCR—still underwent ALND, showing that, unlike in cN1 disease, response-adapted de-escalation has not yet entered routine clinical practice in cN2/N3 breast cancer. The authors also reported that breast pCR and ypN0 were each associated with improved survival (HR 0.31 and 0.39, respectively). A Korean nomogram study of cN2/N3 disease (*n* = 276) [18] reported 49.3% axillary pCR and an area under the curve (AUC) of 0.90 for predicting it by combining imaging with subtype, though external validation is needed. A large unselected Indian series of all subtypes (*n* = 793, of whom 262 were cN2) [19] found lower axillary pCR (58.7% in cN1 and 36.6% in cN2) than the HER2+/TNBC-specific series above, illustrating the subtype-driven heterogeneity that limits pooled estimates; this comparison is confounded by cohort era, staging method, and inclusion of HR-positive disease, and should not be read as evidence of population-level variation in pCR. A US series predominantly comprising cN3 disease (*n* = 521, 73.3% cN3) [20] reported 34.2% axillary pCR (19.4% combined breast/axillary pCR); axillary pCR independently predicted longer event-free survival (HR 0.22, *p* < 0.0001) and TNBC predicted worse EFS (HR 1.74, *p* = 0.008), though distant metastasis still occurred in 29.4% despite response.

Together, these series confirm a consistent, subtype-driven signal: roughly one in three cN2/N3 patients achieves nodal pCR overall, and up to three in four do so when breast pCR is also achieved—approaching the rates that historically justified SLNB/TAD de-escalation in cN1 disease. An important caveat applies to all: pCR was reported after cALND (a fully dissected axilla), not after de-escalated surgery, so a high rate of biological response does not by itself establish that responders can be identified accurately enough with a less extensive procedure to safely reduce surgery.

## 4. Can cALND Be Replaced by TAD/SLNB After an Excellent Radiological Response?

This question can be separated into biological plausibility (do cN2/N3 tumours respond well enough and often enough?) and technical eligibility (can responders be identified with an acceptably low FNR using less extensive surgery?). The evidence for each differs substantially.

### 4.1. Biological Plausibility

As discussed earlier, axillary pCR is achieved in roughly one-third of patients with HER2-positive or triple-negative cN2/N3 disease overall and in up to three-quarters of those who also achieve breast pCR [13,16,17,18,19,20]—the latter approaching the rates observed in cN1 disease in which SLNB and TAD were initially validated. Furthermore, the morbidity associated with cALND is substantial and increases with the extent of axillary surgery, with lymphoedema and shoulder dysfunction occurring in approximately 20–30% of patients [35]. Collectively, these findings suggest that, on biological grounds, cN2/N3 disease is an increasingly compelling candidate for axillary surgery de-escalation.

### 4.2. Technical Eligibility

In validation studies conducted in largely cN1 populations, a prospective multicentre TAD registry (*n* = 548) [8] demonstrated that TAD combining SLNB with marked lymph node biopsy (MLNB) is feasible in routine clinical practice, achieving a detection rate of 86.9% and an FNR of 4.3% (2 of 46; 95% CI 0.5–14.8) compared with 7.2% (8 of 111) for MLNB alone. The 548 figure is the number of patients in whom a clip was placed; only 46 underwent TAD followed by completion ALND and were therefore informative for FNR, so this widely quoted estimate rests on a small denominator. By contrast, a pooled analysis of 3398 patients reported an FNR of 13% and an identification rate of 91% for SLNB alone after NST [36], while a separate pooled analysis of 13 studies including 521 patients undergoing TAD reported an FNR of 5.2% [37]. Collectively, these data suggest that TAD provides more accurate axillary staging than SLNB alone following NST, although the available evidence is derived predominantly from patients with cN1 disease and should not be automatically generalised to those with true cN2/N3 axillary disease.

Until recently, the only individually reported cN2 subgroup was that of ACOSOG Z1071 [4], in which SLN surgery was attempted in 38 cN2 patients with a detection rate of 89.5%; of the 26 who had at least two sentinel nodes excised, 12 achieved nodal pCR, leaving 14 with residual disease in whom the false-negative rate was 0% (95% CI 0–23.2%). An upper confidence bound of that width makes the result hypothesis-generating only, and cN3 disease was excluded from the trial altogether. A further limitation applies to cN2b/cN3a/cN3b/cN3c disease: Level I/II SLNB/TAD does not sample the internal mammary, infraclavicular or supraclavicular compartments that define or contribute to these substages, so even a low-FNR axillary procedure would leave the highest-stage basin histopathologically unassessed. This has direct implications for RNI field design. In patients with initial cN3a (infraclavicular), cN2b or cN3b (internal mammary), or cN3c (supraclavicular) disease, comprehensive RNI covering the involved compartments remains necessary regardless of the pathological response in level I/II nodes. This is because a negative TAD/SLNB result in level I/II, although reassuring, cannot exclude residual disease in the nodal compartment that originally defined the cN2b or cN3 stage. It follows, however, that cALND does not address that compartment either. The case for retaining dissection in cN3a or cN3c disease therefore rests on the level I/II axilla itself rather than on the substage-defining basin, which is treated by irradiation whichever axillary procedure is performed.

Pre-surgical imaging is also insufficiently accurate to establish complete nodal response with a high level of certainty. Vacuum-assisted biopsy (VAB) of the breast, used to assess breast pCR—itself a powerful predictor of axillary pCR—has an FNR of 18–50% in unselected patients, falling to 3–5% with favourable imaging and ≥6 cores [38,39].

A contemporary imaging comparison including 101 locally advanced breast cancer (LABC) cases post-NST [21] confirms the limitations of pre-surgical imaging: Ultrasound had the highest sensitivity (71%) and positive predictive value (PPV) (76%); PET-CT had the highest specificity (73%) and negative predictive value (NPV) (70%); magnetic resonance imaging (MRI) performed worst overall; and radiological–pathological concordance varied by nodal stage (cN3 OR 6.2 vs. cN1, *p* = 0.006) and absence of lymphovascular invasion (OR 9.2, *p* = 0.008). No modality was accurate enough to replace surgical staging, and complete imaging response should not preclude surgical axillary evaluation. In practical terms, none of ultrasound, PET-CT, or MRI currently achieves an NPV high enough to serve as a stand-alone surrogate for surgical staging in cN2/N3 disease. No cN2/N3-specific minimally invasive nodal restaging algorithm currently exists.

A further, disease-specific barrier is axillary burden itself: Unlike cN1 disease, where a single clipped node drives TAD’s low FNR, cN2/N3 disease often involves multiple matted or fixed nodes that are rarely all clipped before NST, creating ambiguity about which node was sampled and whether the specimen represents the pre-treatment burden. SLNB without validated clipped-node retrieval is therefore widely considered unacceptable in a cN2/N3 axilla, and even TAD’s reliability in this multi-node setting is unproven. This gap has now begun to close. A prospective single-centre trial designed specifically to test TAD in advanced nodal disease (*n* = 55 analysed; higher nodal burden defined as cN1 with more than three involved nodes, cN2 or cN3, of whom 53% were cN2 or above) performed TAD followed by completion ALND in the same operation [22]. Technical success was 96%, with the clipped node identified in 100% and at least one additional sentinel node in 96%; axillary pCR occurred in 49%, and among the 27 patients with residual nodal disease, TAD returned an FNR of 0% (95% CI 0–13%) with an NPV of 100%. The same cohort permitted the components of the procedure to be separated: SLNB alone would have yielded an FNR of 11.1% and clipped-node excision alone yielded 18.5%, the latter appreciably higher than in cN1-predominant series and attributed by the authors to the difficulty of identifying the representative node among multiple abnormal nodes. The study also speaks to the matted-node problem raised above, in that marking only the most representative pathological node, rather than every involved node, proved sufficient. Nearly half this cohort was AJCC cN1 by fixation criteria yet carried more than three involved nodes—a presentation common in practice, which shares the multi-node technical problem of ambiguity about which node was sampled and whether it represents the pre-treatment burden, without meeting the matting or fixation criteria for cN2a. Because such disease lies wholly within the level I/II basin, the anatomical argument developed throughout this review applies to it in full, and arguably more directly than to matted cN2a; conversely, the prospective evidence summarised below is correspondingly less confined to AJCC-defined cN2/N3 than its description as advanced nodal disease might suggest. These are the first prospective TAD accuracy data in this population, and they favour the procedure, but they do not settle the question: The FNR denominator is 27 patients, so the upper confidence bound of 13% still exceeds the conventional 10% threshold; the FNR was not reported separately for the cN2/N3 subgroup; and the findings derive from a single centre, three experienced surgeons, a magnetic-seed with superparamagnetic iron oxide (SPIO) and blue-dye technique, and an exclusively Asian population. Read alongside the cN2 subgroup of ACOSOG Z1071, the prospective evidence base can now be stated precisely. A de-escalated axillary procedure has been evaluated against completion ALND, in the same operation, in 87 patients with advanced nodal disease: 53 successful TADs in the present trial and 34 cN2 patients in Z1071 in whom a sentinel node was identified. Of these 87, only 41 had residual nodal disease and were therefore informative for false-negative rate—27 and 14, respectively—since a patient achieving nodal pCR has no true positive to miss. None of the 41 were falsely staged, giving a pooled FNR of 0% (95% CI 0–8.6%), the first estimate in this population for which its upper bound falls below the conventional 10% threshold. This is an exploratory descriptive aggregation, not a formal meta-analytic estimate. That figure should be read with care: the two studies used different procedures, Z1071 excluded cN3 disease altogether, and a single additional false negative would move the pooled rate to 2.4% (95% CI 0.1–12.9%), losing the threshold again. TAXIS [14,15]—the only randomised trial positioned to provide confirmatory evidence—excludes the most extensive presentations (cN2b, cN3c).

**Figure 1:**
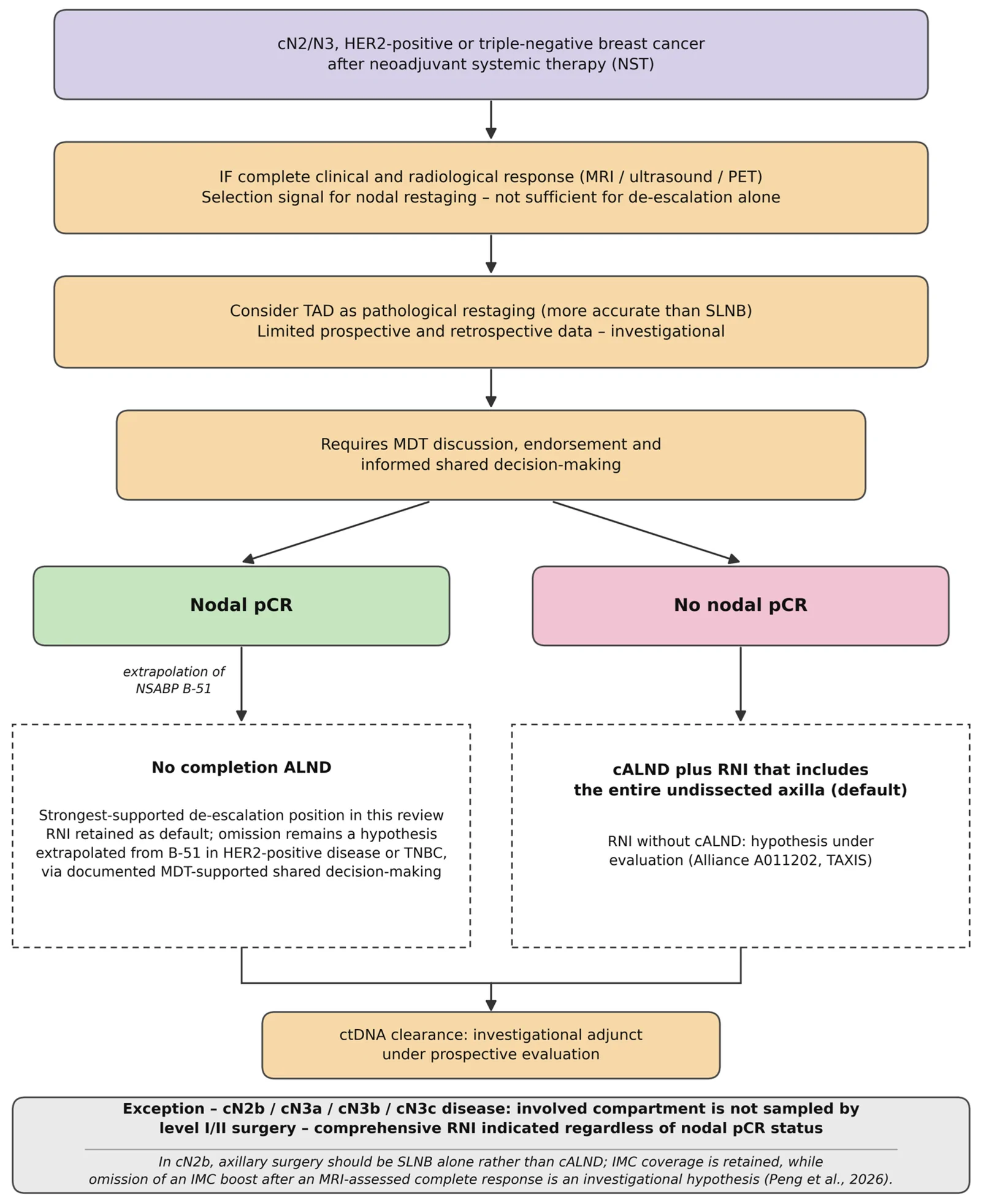
Proposed response-adapted framework for the consideration of axillary de-escalation in cN2/N3 HER2-positive or triple-negative breast cancer after neoadjuvant systemic therapy. Entry to the pathway requires a complete clinical and radiological response on MRI, ultrasound or PET; this is a selection signal for nodal restaging rather than sufficient grounds for de-escalation in itself. TAD is preferred to SLNB alone as the restaging procedure because it is the more accurate of the two, although the prospective and retrospective data supporting it in this population remain limited and its use here is investigational, requiring multidisciplinary team (MDT) discussion, endorsement and informed shared decision-making. Where nodal pCR is confirmed, completion ALND is omitted; this is the de-escalation position with the strongest support in this review, since the axilla is then serving as a restaging rather than a therapeutic field. RNI is retained by default in that setting, and its omission may be considered only as a hypothesis extrapolated from NSABP B-51/RTOG 1304, in HER2-positive disease or TNBC, through documented MDT-supported shared decision-making. Where nodal pCR is not achieved, cALND plus RNI covering the entire undissected axilla remains the default, with RNI without cALND a hypothesis under evaluation in Alliance A011202 and TAXIS; the asymmetry between the two branches is deliberate, since residual disease is demonstrable whereas confirmed pCR is not. Clearance of circulating tumour DNA is shown as an investigational adjunct arising from both branches, under prospective evaluation and not a basis for de-escalation in either. The exception bar applies throughout: In cN2b, cN3a, cN3b and cN3c disease the compartment defining the stage is not sampled by a level I/II procedure, so comprehensive RNI remains indicated irrespective of nodal pCR status. In cN2b, the axilla is clinically uninvolved and axillary staging should be by SLNB alone rather than cALND; internal mammary coverage is retained, while omission of an internal mammary boost after an MRI-assessed complete response is an investigational hypothesis [40]. This figure is a framework for structuring discussion and prospective evaluation, not a validated treatment algorithm. Colour and outline style are used only to distinguish the two response-defined branches (green, nodal pCR; pink, no nodal pCR) and to mark positions that are hypothesis-generating rather than validated (dashed outlines); neither carries any further meaning.

**Real-World Oncological Outcome Data.** The principal directly applicable recurrence evidence in this population comes from the prospective MARI-protocol registry (*n* = 218, extensive nodal disease defined as four or more involved axillary nodes on FDG-PET/CT, July 2014 to December 2021) [23], in which axillary treatment was adapted to response: patients achieving a pCR in the excised MARI node received locoregional radiotherapy alone, whereas those with residual MARI-node disease underwent cALND plus radiotherapy. Forty-seven percent (103/218) achieved ypN0 of the MARI node and had cALND omitted. At a median follow-up of 44 months (IQR 26–62), axillary recurrence after the de-escalated, radiotherapy-alone strategy was 2.9% (3/103), with five-year invasive disease-free survival of 89% (95% CI 83–96) and overall survival (OS) of 95% (95% CI 91–99); in the residual-disease group treated with cALND plus radiotherapy (115/218), the axillary recurrence rate was 3.5% (4/115). These two groups differ systematically in both prognosis and treatment received, and their outcomes should not be read as a comparison between surgical strategies; the registry’s reassurance lies instead in the low absolute axillary-recurrence rate achieved when cALND was omitted in MARI-node responders. Now reported in full, and with an eligibility criterion—four or more involved axillary nodes—that genuinely represents high nodal burden rather than a cN2/N3 subgroup nested within a predominantly cN1 cohort, it is one of only three series discussed here to report an axillary-recurrence numerator and denominator, rather than surrogate pCR or FNR endpoints, after selective ALND omission in this population. Two features of the cohort bear directly on the arguments developed in this review. First, 39% (85/218) had extra-axillary nodal disease on FDG-PET/CT, involving the periclavicular or parasternal chains, which no level I/II axillary procedure samples; the reported axillary recurrence rate therefore describes the axilla specifically, in a population in which a substantial minority had disease outside it. Second, eligibility was defined by a count-based imaging criterion rather than by AJCC subcategory, so the cohort maps onto cN2/N3 disease approximately rather than exactly. Its remaining limitations should temper the weight it carries: it is a non-randomised, single-arm, single-institution registry in which patients were allocated to radiotherapy alone by response rather than at random; the excised MARI node may not represent the biology of the entire axilla; the 44-month median follow-up remains short of the conventional 5-year oncological benchmark; the cohort mixes HER2-positive, TNBC, and HR-positive/HER2-negative disease (36%, 25%, and 39%, respectively, the last falling outside this review’s scope) rather than reporting subtype-specific outcomes; and, spanning 2014–2021, it reflects an earlier and heterogeneous mix of systemic-therapy eras rather than current dual anti-HER2 or pembrolizumab-based regimens. These are hypothesis-generating findings that warrant prospective confirmation, not a validated basis for the response-adapted strategy set out in Figure 1.

A second cohort restricted entirely to advanced nodal disease has since been reported and, together with the MARI registry, constitutes the directly applicable recurrence evidence in this setting. In a single-institution series of 217 consecutive patients with biopsy-confirmed cN2 (*n* = 142) or cN3 (*n* = 75) disease treated with NAC between 2016 and 2024 [24], axillary management was heterogeneous: 122 patients (56.2%) underwent upfront cALND, 60 (27.6%) underwent sentinel node surgery alone, and 35 (16.1%) underwent sentinel node surgery followed by completion cALND. Nodal pCR was achieved in 66 of 142 cN2 patients (46.5%) and 39 of 75 cN3 patients (52.0%), rates consistent with the nodal-response data reviewed above. At a median follow-up of 46 months, axillary recurrence occurred in four patients (1.8%), all of whom had undergone upfront cALND; there were no axillary recurrences among the 60 patients treated with sentinel node surgery alone (0 of 60) or the 35 treated with sentinel node surgery plus completion cALND (0 of 35). Those four axillary events sat within a larger total of nine locoregional (4.2%) and 30 distant (13.8%) recurrences, and crude recurrence of any type still occurred in 6.7% of the sentinel-node-alone group, 14.3% of the sentinel-node-plus-cALND group and 24.6% of the upfront-cALND group (*p* = 0.010); the absence of axillary recurrence after de-escalated surgery therefore describes axillary control specifically and not freedom from recurrence overall. Five-year overall survival did not differ significantly across the three groups (75.9% sentinel node surgery alone, 87.5% sentinel node surgery plus cALND, 85.3% cALND; *p* = 0.056), whereas five-year recurrence-free survival was highest after sentinel node surgery alone (88.8% vs. 73.9% and 69.7%; *p* = 0.022).

Two features of that series govern how much weight it can carry. First, selection was overt and acknowledged by its authors: Patients undergoing sentinel node surgery alone had markedly lower rates of extranodal extension (5.3% vs. 31.3% for cALND within the cN2 subgroup) and lymphovascular invasion, more favourable imaging response, and lower residual nodal burden, so the between-group recurrence comparison reflects prognosis at least as much as extent of surgery. The numerically lower overall survival in the de-escalated group despite its superior recurrence-free survival is a further reminder that these subgroups are not exchangeable, and the observation that recurrence was highest after cALND should not be read as evidence that more extensive surgery is harmful. Second, 92.6% of the cohort received adjuvant radiotherapy with no difference between surgical subgroups, so—as in NEOSENTITURK—the axillary control observed reflects a combined surgical and radiotherapeutic strategy rather than surgical de-escalation in isolation. No completion cALND was performed in the sentinel-node-alone group, so the series contributes no FNR data and cannot address the technical-eligibility question. Its contribution lies elsewhere: it is the first cN2/N3-restricted cohort to report zero axillary recurrences among patients selected for de-escalation, with 95 patients undergoing sentinel node surgery of whom 60 had no completion dissection—a denominator comparable to that of the MARI-protocol registry.

The same series also supplies the first cN2/N3-specific data on how reliably a complete axillary imaging response predicts nodal pCR. Of 44 patients with complete radiological response in the axilla (26 initially cN2, 18 cN3), 43 (97.7%) proved to have nodal pCR at surgery; the single discordant patient had ypN1a disease and had presented with cN2a. This is a considerably more encouraging figure than the imaging negative predictive values discussed above and, if reproduced prospectively, would materially change the calculus of imaging-based selection. It must, however, be read with care: Response was assessed retrospectively from documented radiographic reports rather than by a prespecified imaging protocol, the denominator is small, and patients with a complete imaging response were preferentially routed to sentinel node surgery, so the estimate describes a group already enriched for response. It supports complete imaging response as a selection signal for pathological restaging, not as a substitute for it.

A third cohort confined entirely to advanced nodal disease addresses the same question in a larger and differently selected population. Of 1531 patients with cN2 or cN3 disease who underwent surgery after NAC at a Korean tertiary centre between 2008 and 2021, exclusion of those with a positive sentinel node and those undergoing cALND without prior sentinel node surgery left 432 sentinel-node-negative patients, of whom 297 had sentinel node biopsy alone and 135 proceeded to completion cALND [25]. The initial nodal stage was cN2 in 148 patients (34.3%) and cN3 in 284 (65.7%). At a median follow-up of 44 months, the overall axillary recurrence rate was 2.3% (10 of 432). Five-year axillary recurrence-free survival was 97.3% after sentinel node biopsy alone and 92.9% after completion cALND (*p* = 0.415), with no significant difference in regional recurrence-free survival (96.0% vs. 95.8%, *p* = 0.689) or overall survival (94.2% vs. 87.6%, *p* = 0.972); propensity-score matching of 124 patients per group left these findings unchanged (five-year axillary recurrence-free survival 99.2% vs. 96.6%, *p* = 0.823). Two features give this series particular relevance here, and they point in opposite directions. It is the only cohort discussed in this review to report outcomes according to extra-axillary involvement: 180 patients had internal mammary, and 98 had supraclavicular nodal metastases, of whom only four and 13, respectively, underwent surgical dissection of those compartments, and a subanalysis found no difference between the two axillary strategies either before or after matching. This is the only direct, if retrospective, evidence bearing on the unsampled-compartment problem set out above. Against that, 6 of the 135 patients (4.4%, 95% CI 1.6–9.4%) who proceeded to completion cALND despite a negative sentinel node were found to harbour further nodal metastases, and 5 of those 6 had four or more involved nodes. This is not a false-negative rate in the conventional sense, since sentinel-node-positive patients were excluded from the cohort by design, but it is a direct measure of the residual burden that a negative sentinel node can conceal in a cN2/N3 axilla, and it tempers the reassurance the recurrence data otherwise offer. The remaining limitations are those of the design: retrospective, single-centre, surgeon-determined allocation; a 2008–2021 treatment era predating contemporary dual anti-HER2 blockade and immunotherapy-containing regimens; and radiotherapy in 95.6% of patients so that axillary control here too reflects a combined surgical and radiotherapeutic strategy rather than surgical de-escalation alone.

An NCDB analysis of 22,156 women with cN2/N3 disease from 2004–2013 [26] found that cALND was associated with improved OS versus SLNB after adjustment (HR 0.68, *p* < 0.001), but this cohort predominantly underwent upfront surgery (only 39.7% received NST) and was vulnerable to confounding by indication. A more recent, more advanced NCDB cohort (*n* = 7167, cN2b–N3c, adjuvant-RT-treated) [27] partially tempers this: after inverse probability of treatment weighting (IPTW) adjustment, ALND was not associated with improved OS versus SLNB (HR 0.99, *p* = 0.91)—directly contrasting the unadjusted signal noted above. This cohort is distinctive in lying entirely within the high-burden nodal categories, and is in practice an N3 dataset (cN2a is excluded; 7% cN2b, 19% cN3a, 24% cN3b, 19% cN3c and 31% cN3 not otherwise specified), whereas the other series discussed here are cN1–3 cohorts in which cN2/N3 patients form a minority. Two constraints cap the weight it can carry: neoadjuvant exposure and pathological nodal response were not characterised, so de-escalation after response cannot be distinguished from de-escalation in unresponsive disease; and overall survival was the sole endpoint, with no axillary-recurrence or disease-free survival data, which is insensitive to precisely the locoregional failures that axillary surgery is intended to prevent. Because both are retrospective registries with confounding in opposite directions, the survival benefit of more extensive surgery in cN2/N3 disease remains genuinely unresolved.

A further NCDB analysis restricted to cN3b disease (*n* = 4236, 2012–2021) [41] addresses the one subgroup in which the internal mammary component is, by definition, unsampled by any axillary procedure. Use of SLNB alone rose from 8.5% of these patients in 2012 to 35.1% in 2021 (*p* < 0.0001); among those receiving neoadjuvant chemotherapy the nodal pCR rate was 24.9%, and overall survival for patients undergoing SLNB or SLNB plus ALND with nodal pCR did not differ significantly from that after ALND alone (*p* = 0.22 and *p* = 0.57, respectively), leading the authors to conclude that de-escalation may be considered in well-selected cN3b patients responding excellently to NST. The same reservations apply—retrospective selection, no locoregional-recurrence endpoint, and no account of radiotherapy fields—and the finding bears only on patients in whom nodal pCR was pathologically confirmed, not on those selected by imaging response alone.

A Mayo Clinic series enrolled 602 patients with biopsy-proven cN1–3 disease [28] and reported a 2-year freedom-from-regional-recurrence rate of 99.1% (SLN surgery) versus 96.4% (ALND, *p* = 0.10, not significant). This is indirect rather than direct evidence for the present question, for two reasons. First, selection for SLN surgery was significantly associated with a lower presenting nodal category—that is, with cN1 rather than cN2/N3—so the de-escalated group is enriched for precisely the patients in whom de-escalation is already validated. Second, the published recurrence result, a single regional recurrence among the 159 patients treated with SLN surgery alone, is not stratified by initial cN category, and no cN2/N3-specific numerator and denominator can therefore be extracted from it. A subsequent review has in fact described those 159 patients as pre-neoadjuvant cN1; until that is reconciled against the primary report, this series should not be counted as evidence bearing specifically on cN2/N3 disease. Follow-up was also short.

The prospective NEOSENTITURK MF-1803 registry (*n* = 976, 37 centres) [29] is supportive but, on close reading, indirect. Its cohort was predominantly cN1: 792 patients (81.1%) were cN1 and only 184 (18.9%) were cN2/N3, comprising 153 cN2 and 31 cN3. Higher nodal burden was nonetheless not rare, since among the 898 patients for whom imaging node counts were available, 333 (37.1%) had three or more metastatic or suspicious axillary nodes. Ipsilateral axillary recurrence was very rare in absolute terms, with three events across the whole cohort, and similar between TAD and SLNB (1 of 356 vs. 2 of 620, 0.3% vs. 0.3%). Those three recurrences are reported by surgical procedure rather than by initial nodal category, however, so no axillary-recurrence numerator is available for the 184 cN2/N3 patients. The registry’s excellent overall axillary control therefore cannot be assumed to apply specifically to them, and for the same reason those patients cannot legitimately be entered into a pooled high-nodal-burden recurrence analysis. All patients also received regional nodal irradiation encompassing the axilla, so the control observed reflects a combined surgical and radiotherapeutic strategy rather than surgical de-escalation alone. What the registry does report by nodal category is survival, and here initial burden retained its prognostic weight: cN2/N3 stage was independently associated with lower 5-year disease-specific survival than cN1 (96.8% vs. 98.7%, *p* = 0.03) even within this de-escalated cohort, indicating that pre-treatment nodal burden is not fully erased by favourable response; a separate, subtype-specific caution emerges from the OPBC-07/microNAC cohort [42], as discussed below. Its cN2/N3 subgroup was likewise not separately analysed for FNR, so it does not resolve the central technical-eligibility question.

A TNBC-specific NCDB analysis restricted to patients receiving neoadjuvant chemo-immunotherapy (*n* = 4336 cN1–3, of whom 678 were cN2/N3) [30] used ypN0 as its selection criterion for de-escalation and found that SLNB yielded 5-year OS comparable to that of ALND (90.1% vs. 93.6%, *p* = 0.99, with an identical 48-month restricted mean survival time), with no significant survival detriment on multivariable analysis (aHR 1.19, 95% CI 0.54–2.61, *p* = 0.67). This is again a mixed cN1–3 cohort, although one in which the cN2/N3 subgroup was analysed separately; locoregional recurrence was not an available endpoint, so it contributes survival rather than axillary-control data. Because selection again relied on confirmed nodal pCR rather than an unconfirmed excellent response, it strengthens the pCR-scenario evidence discussed later more than it resolves the FNR question addressed here.

**Practical Implication.** This evidence base is tiered rather than uniform and should be read as such. Only three cohorts yield an axillary-recurrence estimate drawn exclusively from advanced nodal disease: the MARI-protocol registry [23], the single-institution cN2/N3 series of Rangan and colleagues [24], and the sentinel-node-negative cN2/N3 cohort of Shin and colleagues [25]. None of the three provides FNR data. The remaining cohorts [27,28,29,30,41] are either cN1–3 series in which cN2/N3 patients form a minority whose recurrence events are not reported separately, or registry analyses with no locoregional endpoint at all, and they are therefore supportive rather than directly applicable. Read with that hierarchy in view, the consistency of a reassuring oncologic signal across multiple independent cohorts spanning different populations, institutions, and selection criteria (Table 4) represents accumulating, if still non-randomised and hypothesis-generating, evidence that axillary surgical de-escalation in cN2/N3 disease may not carry the survival penalty once assumed. This narrows, without closing, the gap separating cN2/N3 from the validated cN1 pathway; it does not resolve the separate, still-open question of whether TAD/SLNB can identify true nodal pCR with an acceptably low FNR in this population. Outside a clinical trial, TAD/SLNB should not yet be considered a formally validated substitute for cALND in cN2/N3 disease, even after an excellent radiological response. It is equally important not to overstate what cALND itself offers. The unadjusted signal favouring more extensive surgery reported in the earlier NCDB analysis [26] was not reproduced after adjustment in the more recent cohort [27], so its survival advantage in this population remains unproven. The more informative observation, however, is not simply that a benefit from cALND has never been demonstrated, since absence of proof is not proof of absence, but that no oncological penalty has yet emerged when surgery is de-escalated in carefully selected responders, across cohorts differing in country, era, institution and selection method. That consistency shifts the question from why cALND might be omitted to what incremental oncological benefit it delivers once nodal pCR has been documented and modern systemic therapy, with or without RNI, has already treated the disease. The available data do not answer that question with certainty, but they increasingly suggest the increment is small. What they do not do is establish equivalence: the cohorts reporting no penalty are selected, non-randomised and largely irradiated, and the false-negative rate on which any claim of documented nodal pCR depends is itself unvalidated in this population. cALND therefore remains the conservative, guideline-supported standard, but as a default held under genuine uncertainty rather than as a procedure of demonstrated oncological value. A patient and an MDT weighing a de-escalated approach outside a clinical trial are therefore doing so under substantial evidentiary uncertainty, which provides a rational basis for prospective clinical equipoise but does not itself establish it; any such decision should be documented as an individualised, MDT-supported shared decision, ideally with clipped node retrieval, dual mapping, and a low threshold for completion ALND if fewer than three nodes (including the clipped node) are retrieved.

**A Technical Hierarchy.** Where a de-escalated axillary procedure is undertaken in high-burden nodal disease, TAD—retrieval of the biopsy-proven, marked node together with the mapped sentinel nodes—remains the reference technique, because its accuracy derives from documented removal of the node known to have contained disease rather than from mapping alone [8]. Where no marked node is available, or it cannot be retrieved, and SLNB alone is performed, the number of sentinel nodes recovered becomes the principal modifiable determinant of accuracy. In arm C of SENTINA, the false-negative rate was 24.3% when a single sentinel node was removed and 18.5% when two were removed, against 14.2% for the arm overall [5]; in ACOSOG Z1071 it was 21.1% with two sentinel nodes but 9.1% when three or more were examined, and 10.8% with dual-agent mapping versus 20.3% with a single agent [4]. Retrieval of at least three sentinel nodes with dual-tracer mapping should therefore be treated as a minimum technical requirement, not an aspiration, wherever SLNB alone is relied upon. The hierarchy is reinforced by the prospective high-burden data discussed above [22], in which TAD returned an FNR of 0% against 11.1% for SLNB alone and 18.5% for clipped-node excision alone within the same patients, the gap widening rather than narrowing as nodal burden increased. Two qualifications nonetheless remain: Retrieval of three or more sentinel nodes brings the false-negative rate only to the margin of the conventional 10% threshold and does not reproduce the accuracy of marked-node retrieval, so node count is a partial substitute for TAD rather than an equivalent to it; the SENTINA and Z1071 node-count gradients themselves derive from cN1-predominant cohorts and have not been re-measured in a cN2/N3 axilla, where the multi-node burden described above makes any individual mapped node less likely to represent the pre-treatment disease.

**A Scoping Caveat: cN2b.** The arguments developed in this section concern whether an axilla that was involved at presentation can be de-escalated, and on that reading they do not apply to cN2b. Because cN2b is defined by internal mammary involvement in the absence of clinically evident axillary disease, the axilla is cN0 at presentation, and cALND is not indicated at baseline; its omission there is a matter of staging definition rather than of de-escalation, and does not depend on the depth of radiological response. Axillary staging in cN2b accordingly follows the clinically node-negative pathway—SLNB after NST rather than TAD, since no involved axillary node has been biopsied and marked—and SLNB alone is sufficient, with completion ALND reserved for a positive sentinel node on the same criteria applied to any cN0 axilla. The technical benchmark that matters here is therefore the post-NST SLNB false-negative rate in clinically node-negative disease, not the cN1-converting figures discussed above. The unresolved question in cN2b is not axillary surgery but coverage of the internal mammary chain, addressed later in this review.

## 5. Hypothesis-Generating Immunological Considerations

Regional lymph nodes are active secondary lymphoid organs where tumour-specific T-cell priming occurs, which is relevant to TNBC (where neoadjuvant pembrolizumab/KEYNOTE-522 [43] is now standard) and increasingly to HER2-positive disease. Preclinical work shows that tumour-draining lymph nodes harbour T-cell populations required for effective PD-1/PD-L1 blockade, and resecting the draining node abolished checkpoint-induced tumour regression in mouse models [44]; immunologically active regional nodes have also been associated with improved survival in early-stage TNBC [45]. This preclinical mechanism has been invoked to explain an exploratory clinical signal in cN1 disease: In the B-51/RTOG 1304 TNBC subgroup, an exploratory analysis showed that RNI was associated with numerically worse outcomes than no RNI (HR 2.30, 95% CI 1.00–5.25). However, whether surgical removal similarly impairs checkpoint-inhibitor efficacy in humans is entirely unproven, with no direct clinical data on this in breast cancer. This remains a preclinical, hypothesis-generating consideration, not evidence against nodal clearance or a clinical argument for reducing axillary surgery.

In the context of cN2/N3 disease, this preclinical hypothesis does not currently justify de-escalation on immunological grounds alone, as no clinical data link surgical extent to immunotherapy efficacy in breast cancer, but it adds a third, correlative dimension, alongside pCR-based biological plausibility and FNR-based technical eligibility, that future cN2/N3 trials should incorporate as an exploratory immune endpoint rather than as a basis for current practice.

## 6. The pCR Scenario: Can cALND Be Omitted, Replaced by RNI, or Omitted Together with RNI?

NSABP B-51/RTOG 1304 [10] is the only randomised trial testing RNI omission after nodal pCR. It enrolled 1641 cT1–3N1M0 patients converting to ypN0, randomised to RNI versus no RNI; the 5-year recurrence-free interval exceeded 91% in both arms (HR 0.88), but the protocol explicitly excluded cN2, cN3, and cT4 disease. B-51 asked whether surgically confirmed ypN0 patients need added RNI; the cN2/N3 question is upstream, namely, whether axillary surgery itself can be reduced at all, and B-51 offers no direct evidence on that [46]. Extrapolation is further limited by three factors: higher pre-treatment nodal burden independently predicts recurrence even after pCR (in 292 patients achieving pCR/near-pCR, cN3 status independently predicted recurrence in both groups, *p* = 0.003 after pCR and *p* = 0.036 after near-pCR, with 5-year recurrence-free survival of 94.6% after pCR and 85.6% after near-pCR [31]); ypN0 in B-51 was confirmed predominantly by SLNB rather than TAD; no trial has randomised cN2/N3 patients with confirmed pCR to RNI-alone versus surgery. For initially extensive axillary disease—that is, cN2a and axillary cN3 rather than the cN2/N3 category generically, since in cN2b the axilla is uninvolved at presentation and cALND is not indicated at baseline—cALND plus RNI therefore remains the current conservative, guideline-supported standard in the absence of cN2/N3-specific prospective evidence; the alternatives below are extrapolations, not validated practice.

Three positions are in play once nodal pCR is confirmed by TAD or SLNB: cALND plus RNI (the conservative default); RNI alone, omitting cALND; or omission of both. RNI in this context is not treating known disease but hedging against the incompletely validated FNR of TAD/SLNB in cN2/N3 disease. It is therefore most defensible where confidence in the pCR result is lowest (high nodal burden, non-HER2-positive subtype, or no confirmed clipped-node retrieval), but it also concedes the review’s central problem: “confirmed pCR” cannot yet carry the same weight in cN2/N3 disease as in cN1.

Whether full omission is defensible depends on subtype. B-51’s HER2-positive subgroup (57% of the cohort) showed no RNI benefit, and RNI’s own overall-survival benefit is itself debated in its validating trials (NCIC-CTG MA.20: DFS benefit but no OS difference, HR 0.91, *p* = 0.38 [47]; EORTC 22922: similar pattern, p narrowly non-significant [48]). Extrapolated to cN2/N3 HER2-positive disease with confirmed pCR, accepting TAD including SLNB confirmation, which is, if anything, a more conservative threshold than B-51 itself used, this supports full omission as an investigational hypothesis, provided that the B-51 exclusion, the lack of cN2/N3-specific data, and the unvalidated FNR underpinning the pCR confirmation are disclosed. In B-51’s TNBC subgroup, an exploratory analysis with few events, RNI was associated with numerically worse outcomes (HR 2.30 for RNI, 95% CI 1.00–5.25); because the trial’s primary analysis showed no benefit from RNI and this subgroup finding is hypothesis-generating only, it should not be used as a positive rationale for omitting RNI. The extrapolation logic applied above to HER2-positive disease may nonetheless also be examined in TNBC after confirmed nodal pCR, despite this specific population not being tested in B-51. As with HER2-positive disease, this remains a hypothesis for prospective evaluation rather than routine practice: the subgroup analysis was exploratory, its confidence interval is wide with a lower bound approaching 1, and B-51 excluded cN2/N3 disease altogether. Within these constraints, the TNBC result provides no evidence of RNI benefit but equally cannot exclude one, and RNI omission in either subtype should be pursued only through MDT discussion, endorsement, and informed shared decision-making. HR-positive/HER2-negative disease showed a trend favouring RNI that reached nominal significance within this exploratory subgroup analysis (HR 0.41, 95% CI 0.17–0.99 [10]) and, being outside this review’s core scope, offers no basis for omission.

Taken together, confirmed nodal pCR in cN2/N3 disease does not currently support routine RNI omission in either HER2-positive or TNBC subtypes. In both, nodal pCR confirmed by TAD or SLNB supports prospective evaluation of full omission of further axillary treatment, meaning neither cALND nor RNI. This is an extrapolated hypothesis rather than a candidate for routine adoption and should be pursued in either subtype only within a trial or through a fully individualised, documented shared decision; AMAROS [49,50] is not relevant here, since it addresses substituting RT for surgery in the setting of residual disease—and even there, it is only a hypothesis-generating extrapolation from a predominantly upfront-surgery, sentinel-node-positive population—not after confirmed pCR. This hypothesis rests entirely on retrospective cN2/N3 concordance data and extrapolation from B-51 and applies mainly to cN2a and other level I/II-predominant disease, since level I/II TAD/SLNB does not sample the compartments defining cN2b/cN3a/cN3c, where comprehensive RNI remains indicated regardless of pCR status. As detailed in Section 9, the absence of a validated cN2/N3-specific FNR for TAD/SLNB remains the most urgent gap; outside dedicated FNR and RNI-omission trials, this remains a hypothesis for prospective testing, not a routine practice standard. Of the clinical situations considered in this review, however, it is after documented nodal pCR that the case against further axillary treatment is strongest, and within that setting the two components are not equally supported: Omission of cALND rests on the accumulated outcome data reviewed above, whereas additional omission of RNI rests on extrapolation from a trial that excluded this population and is correspondingly more speculative. No residual disease has been demonstrated in the surgically sampled nodal basin, so the axilla is functioning principally as a restaging rather than a therapeutic field, and the residual uncertainty attaches to the accuracy of the restaging procedure rather than to the biology of what might be left behind.

**Figure 2:**
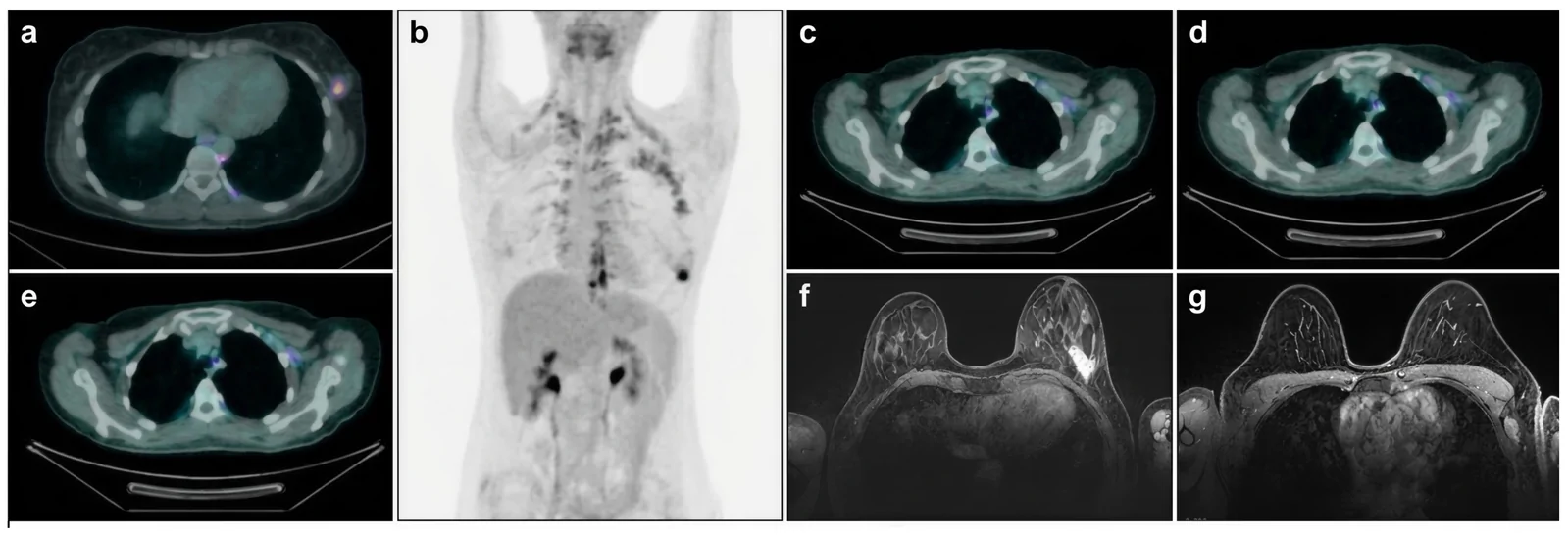
Pre-treatment PET (**a**–**e**) and MRI (**f**) demonstrating clinical staging (cT3N3c) in a 47-year-old woman diagnosed with triple-positive, grade 3 invasive ductal carcinoma. The patient received neoadjuvant systemic therapy (NST) and achieved a complete radiological response on MRI (**g**). Following multidisciplinary discussion and shared decision-making, she underwent breast-conserving surgery (BCS) with targeted axillary dissection (TAD) without completion axillary lymph node dissection (cALND), confirming a pathological complete response (pCR). She subsequently received radiotherapy to the left breast and regional nodal irradiation (RNI), excluding the dissected TAD region. She completed one year of anti-HER2 therapy and is currently receiving letrozole. The patient remains disease-free at 42 months of follow-up. Axillary surgery alone was de-escalated: Comprehensive regional nodal irradiation was retained, consistent with the position taken throughout this review that in cN3c disease the compartment defining the stage is not sampled by a level I/II procedure and comprehensive RNI remains indicated irrespective of level I/II nodal pCR. This is an illustrative clinical case included to show how the considerations discussed above translate into practice; a single favourable outcome is not evidence supporting the oncological safety of de-escalation.

**Shared Decision-Making in Current Practice: A Pragmatic Framework.** In the absence of cN2/N3-specific trial evidence, a pragmatic approach is to bring these cases to the MDT meeting and then discuss directly with the patient, explaining that B-51 excluded cN2/N3 disease and that the extrapolation above is a reasoned hypothesis, not validated evidence. This is weighed against RNI’s known toxicity (lymphoedema on top of surgical risk, brachial plexopathy, radiation pneumonitis, and, for left-sided disease, cumulative cardiac radiation exposure), particularly once nodal pCR is achieved, and the marginal benefit of adding RNI is most uncertain. The decision to add or omit RNI becomes an explicit, documented shared decision rather than a default extension of cN1 trial data. Figure 2 illustrates a clinical case in which axillary surgery, but not regional nodal irradiation, was de-escalated in a patient presenting with cT3N3cM0 HER2-positive breast cancer that responded completely to NST.

## 7. The Non-pCR Scenario: RNI Versus cALND for Residual Axillary Disease

For residual axillary nodal disease, cALND remains the standard while Alliance A011202 (NCT01901094) [9] tests whether axillary RT is non-inferior to cALND (both combined with extended RNI). The trial enrolled only cT1–3N1 patients with a positive sentinel node and excluded cN2/N3 entirely, so its results cannot be directly extrapolated. A SABCS report from the trial [51] found that 30.5% of enrolled patients had additional positive nodes at completion of ALND (38.4% among ypN1mi-only patients), underscoring that occult additional disease is common even in a lower-burden cN1 population, which argues for caution in extrapolating any non-inferiority result upward.

TAXIS (NCT03513614) [14,15] is the only randomised trial open to a portion of the cN2/N3 spectrum, comparing tailored axillary surgery (TAS) alone plus RT versus TAS plus completion ALND plus RT; it excludes cN2b and cN3c disease. A feasibility substudy (*n* = 296, 42.2% NST-treated) [52] showed that TAS was technically feasible with meaningful tumour-volume reduction, but oncological outcomes are not yet mature. An older PET-CT-staged cohort (*n* = 55, cN3) [32] offers a cautionary signal regarding basin-directed RT intensification: only 5.5% failed at the original PET-positive site, and neither RT to PET-positive internal mammary regions (cN3b) nor supraclavicular dose escalation to ≥55 Gy (cN3c) improved 5-year relapse-free or disease-free survival, with distant metastasis being the dominant failure pattern. This suggests that intensifying RNI to imaging-positive basins alone may not be the lever that improves outcomes at this nodal burden.

A further complexity, largely unaddressed by the binary “ALND versus no ALND” framing, is that residual disease is not a single entity: Isolated tumour cells, micrometastases (ypN1mi), macrometastatic disease, extranodal extension, and node count each carry different risk profiles, and real-world practice already omits ALND selectively for low-volume disease such as ypN1mi. This is not merely theoretical: The international OPBC-07/microNAC cohort (1585 patients with residual micrometastases, 50.7% undergoing completion ALND and 79.9% receiving nodal irradiation) [42] reported a 3-year rate of any axillary recurrence of 2.0% (95% CI 1.3–2.9) overall, with no difference by extent of axillary surgery, at a median follow-up of 3.1 years. Tumour biology, however, modified that result: Among patients with triple-negative disease, those who did not undergo completion ALND had a significantly higher rate of any axillary recurrence than those who did (8.7%, 95% CI 4.4–15.0, vs. 2.4%, 95% CI 0.7–6.5; *p* = 0.018), leading the authors to conclude that biology should be taken into account when ALND omission is considered. That signal arises in ypN1mi rather than ypN0 disease and in a predominantly cN1 population, so it does not bear directly on the pCR-based extrapolation developed earlier; it is nonetheless the clearest available counterweight to a permissive reading of the exploratory B-51 triple-negative subgroup and argues for particular caution about de-escalation in triple-negative disease wherever residual nodal disease of any volume is found. A single isolated tumour cell or micrometastasis in an axilla that began as cN2/N3 disease may not, therefore, carry the same risk as an identical finding in a cN1 axilla. Because initial cN2/N3 disease implies a substantially higher pre-treatment nodal burden than cN1, a finding of ypN1mi in this population plausibly reflects a greater volume of residual disease across the undissected nodal field than an identical ypN1mi finding arising from cN1 disease, providing a further reason to be cautious in extrapolating any eventual Alliance A011202 non-inferiority result to cN2/N3. Until specifically studied, ypN0(i+) (residual isolated tumour cells only) or ypN1mi in initially cN2/N3 disease should be regarded as an unvalidated grey zone rather than as being safely manageable with RNI alone by direct extrapolation from cN1 data.

The only large observational dataset that approximates this strategy within the relevant stage range is the NCDB cohort of 7167 women with cN2b–N3c disease discussed earlier, all of whom received adjuvant radiotherapy [27]. In that setting—less extensive axillary surgery combined with radiotherapy covering the undissected nodal field—IPTW-adjusted overall survival after SLNB was not inferior to that after ALND (HR 0.99, *p* = 0.91). This is the closest real-world approximation available of the question Alliance A011202 and TAXIS are designed to answer, and the only such dataset confined entirely to the high-burden nodal categories. Its reassurance is nevertheless bounded by the limitations set out above: Neoadjuvant exposure and pathological nodal response were not characterised, so responders cannot be separated from non-responders; axillary recurrence was not reported, and overall survival is an insensitive endpoint for a locoregional intervention; the 13% who underwent SLNB were selected in ways that inverse probability weighting cannot fully correct. It therefore supports the plausibility of radiotherapy substituting for completion dissection in this population without establishing it.

Until Alliance A011202 and TAXIS reports, and until a cN2/N3-specific residual-disease trial is conducted, ideally stratified by residual burden, cALND (± RNI as dictated by burden and receptor/HER2 status) remains the standard approach for this subgroup in the absence of cN2/N3-specific prospective evidence; in cN2b/cN3b disease, the internal mammary component is unaddressed by axillary surgery of any extent and requires RNI coverage in addition to whatever axillary procedure is performed. The asymmetry between this scenario and the pCR scenario is deliberate. Where nodal pCR has been documented, the argument for omitting further axillary treatment rests on the absence of demonstrable disease; where disease persists, its burden is demonstrable, the relevant randomised evidence is either cN1-derived or only partially applicable, and the triple-negative signal from OPBC-07/microNAC [42] is a direct caution against assuming that residual low-volume disease can be managed without dissection in every subtype. De-escalation after residual disease is therefore plausible but materially less mature than de-escalation after confirmed nodal pCR. Extrapolating an eventual A011202 non-inferiority signal from a cN1, single-positive-node population to a cN2/N3, multi-node-positive population would substitute a lower-risk trial result for a materially different, higher-risk scenario, which is why the extrapolated alternative set out below is offered as a hypothesis for individualised discussion rather than a validated substitute.

## 8. Ongoing and Planned Trials

**Figure 3:**
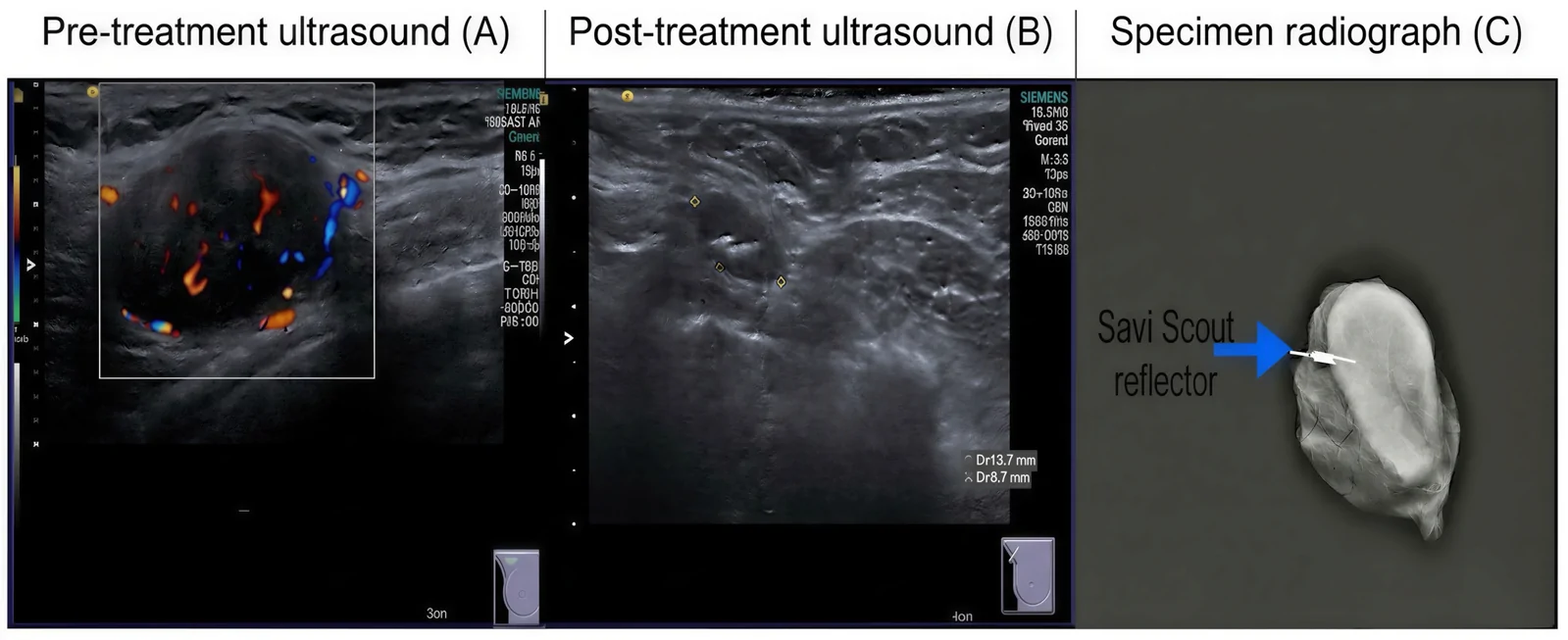
Axillary lymph node response to NST in TNBC. (**A**) Pre-treatment ultrasound shows an enlarged, hypervascular pathological lymph node with increased colour flow Doppler. (**B**) Post-treatment ultrasound demonstrates a complete radiological response with normalised nodal architecture and a localised central hyperechoic SAVI SCOUT reflector. (**C**) Specimen radiograph demonstrating a SAVI SCOUT reflector in a targeted axillary dissection (TAD) sample.

Several prospective initiatives are relevant, though none is yet cN2/N3-specific or definitive: a randomised trial evaluating SLNB accuracy for residual cancer after NST in locally advanced disease (NCT03255577); studies of carbon nanoparticle or indocyanine-green tracing to improve nodal mapping (NCT05241119, NCT06085274); and imaging-based trials aiming to reduce reliance on axillary surgery for systemic therapy decisions in cN2 disease (NCT03857932, NCT04072653, NCT04274946). None is powered for a cN2/N3-specific oncological endpoint. The AXSANA registry (NCT04373655, EUBREAST-03) is also relevant: It is an international prospective cohort of over 6000 patients comparing SLNB, TAD, and ALND in cN+ disease converting to ycN0 after NST. It enrols cN2/N3 patients (13.3% of the marking cohort had ≥4 suspicious nodes at diagnosis) unlike TAXIS, though it is observational rather than randomised and therefore cannot itself validate an FNR. Its initial marking-technique results found that target-lymph-node detection rates varied significantly by marking technique, with clip marking performing worst (radioactive seed 100%, magnetic seed 96.9%, radar marker 96.1%, carbon ink 94.9%, radiofrequency identification (RFID) 90%, clip 89.6%; *p* < 0.001) [53] (Figure 3), a technical finding directly relevant to the FNR of any future cN2/N3-specific TAD/SLNB validation study, since marked-node retrieval failure is itself a source of false-negative results. This is reinforced by a pooled analysis of 17 studies and 1358 wire-free TAD procedures, which reported localisation and retrieval rates of 97% and 99%, respectively, but only 67% concordance between MLNB and SLNB, with omission of MLNB or SLNB estimated to have under-staged the axilla in 15.2% and 5.4% of cases, respectively (*p* = 0.0001) [54]. Together, these data indicate that TAD outperforms SLNB alone for axillary staging in the predominantly cN1 populations in which both have been assessed and that the accuracy of either depends on successful retrieval of the marked node.

A more directly relevant initiative is SenTa2 (NCT05462457, Kliniken Essen-Mitte) [55], a prospective multicentre registry designed specifically to close this gap: It enrols patients with ≥3 initially suspicious lymph nodes, a burden overlapping the cN2/N3 population, who convert to ycN0 after NST, and compares TAD with a subsequent completion ALND in the same patients to directly measure TAD’s FNR in this high-burden setting. Building on the earlier SenTa registry, which reported an axillary pCR rate of 60.3% in 473 cN+ patients but did not itself address FNR in the high-burden subgroup, SenTa2 (estimated enrolment of 150, primary completion in 2027) is, together with the cN2a/N3a-eligible subgroup of TAXIS, among the most direct near-term opportunities to generate FNR data for a meaningful fraction of this population.

## 9. Limitations of the Current Evidence Base

This is a narrative rather than a systematic review; no formal risk-of-bias assessment was applied, and study selection retains an element of author judgement. Most cN2/N3-specific nodal-response and oncological-outcome data are retrospective and susceptible to selection bias regarding who was offered less extensive surgery; prospective technical-validation data remain limited to small cohorts and are not adequately powered for cN2/N3-specific conclusions. No study has reported a prospective FNR of TAD/SLNB restricted to cN2/N3 disease with an adequate sample size. The oncological-outcome studies discussed above are limited by registry-level confounding [26,27] or small, single-institution experiences with short follow-up [28]; NEOSENTITURK MF-1803 [29] did not report axillary-specific recurrence or FNR separately for its cN2/N3 subgroup, which accounted for only 184 of 976 patients (18.9%), and all of its patients received regional nodal irradiation covering the axilla, while the Mayo Clinic series [28] likewise reports its single regional recurrence among 159 patients treated with SLN surgery alone without stratification by initial cN category in a cohort in which selection for SLN surgery favoured cN1 disease. The single-institution cN2/N3 series [24] is limited by its retrospective design, by selection differences between surgical groups that its authors describe explicitly—extranodal extension, lymphovascular invasion, imaging response, and residual nodal burden all differed—by the absence of completion cALND in the de-escalated group, which precludes any FNR estimate, and by the fact that 92.6% of its patients received adjuvant radiotherapy, so its axillary control cannot be attributed to surgical strategy alone. The limitations of the TNBC-specific NCDB study [30] include missing data on locoregional recurrence, incomplete details of the SLNB technique, and the inability to distinguish TAD from SLNB. While comorbidities were adjusted for, some confounding remains, and the relatively short follow-up for the immunotherapy group means that long-term outcomes still need confirmation. Finally, the MARI-protocol registry [23], although now reported in full, remains a non-randomised single-arm study in which allocation to radiotherapy alone was determined by response rather than randomised, so its low axillary-recurrence rate describes appropriately selected responders rather than cN2/N3 disease at large. Furthermore, most included cohorts predate widespread dual anti-HER2 blockade and pembrolizumab-containing TNBC regimens; contemporary pCR rates are likely higher, which may render historical FNR estimates conservative. This remains uncertain, however, since higher-burden disease could behave differently under intensified therapy. Critically, a higher pCR rate does not automatically translate into safer de-escalation: the technical (FNR) and immunological considerations discussed above are independent of the pCR rate and must be resolved on their own terms.

**Clinical Staging Heterogeneity.** cN2/N3 is not a single, biologically uniform category. AJCC subdivides cN2 into cN2a (fixed/matted axillary nodes) and cN2b (internal mammary nodes without axillary involvement) and cN3 into cN3a (infraclavicular), cN3b (internal mammary plus axillary), and cN3c (supraclavicular), categories with plausibly different risks. Clinical N-staging is also method-dependent (palpation, ultrasound, PET, and MRI each differ in accuracy), and pre-NST cytological or histological nodal confirmation was not universal: In the CSBrS-012 cohort, 74.5% of patients underwent node biopsy, meaning approximately one-quarter were staged cN2 on clinical examination and imaging alone [17], a source of potential stage misclassification that could inflate apparent nodal response rates. Future cN2/N3-specific studies should report outcomes stratified by AJCC subcategory and staging method.

**Internal Mammary Chain Involvement.** Isolated IMC involvement is a further source of anatomical heterogeneity not addressed by the pathway in Figure 1. Because level I/II TAD/SLNB does not sample the IMC regardless of axillary nodal status, IMC-positive disease with no or low-volume axillary involvement—which upstages to cN2b—falls outside the axillary-focused de-escalation logic developed above. Primary tumour location bears on the pretest probability of IMC involvement: In a lymphoscintigraphy series of 220 patients, IMC drainage was documented in 17% overall and was significantly more common from lower-quadrant and central tumours (25–29%) than from the upper-outer quadrant (10%), while isolated IMC drainage without axillary drainage was rare (1%) [56]. This pattern is more accurately characterised as location-dependent than strictly medial-versus-lateral, but it confirms that primary tumour location remains a relevant covariate when interpreting a positive IMC finding. A dedicated evaluation of IMC-positive, axilla-negative or low-volume axillary disease represents a further gap in the anatomical framework used here.

Early evidence suggests that the response-adapted logic developed here for the axilla may extend to the IMC through imaging rather than surgical restaging. In a retrospective, IPTW-adjusted series of 208 patients with clinically positive internal mammary nodes at baseline, a postoperative internal mammary radiotherapy boost was associated with improved disease-free survival only in patients without an MRI-assessed internal mammary complete response after NST (HR 0.36, 95% CI 0.14–0.93), whereas no benefit was apparent in those achieving complete response (HR 1.13, 95% CI 0.47–2.74; interaction *p* = 0.042) [40]. Although retrospective, with a median follow-up of only 36 months, this supports prospective evaluation of MRI-assessed internal mammary response as a non-invasive selector for response-adapted irradiation of a compartment in which pathological restaging is not feasible. These considerations converge in cN2b disease specifically. Because the compartment defining the stage is the IMC and the axilla is clinically uninvolved, axillary surgery should be SLNB alone rather than cALND, and IMC coverage is retained by default, while omission of an IMC boost after an MRI-assessed complete response, and more broadly whether comprehensive RNI could be omitted or irradiation restricted to the IMC in such responders, remains an investigational hypothesis requiring prospective evaluation [40]. A corollary applies to field design. If the IMC is the only compartment that defines the stage, and the axilla is both clinically uninvolved and, where TAD/SLNB is performed, pathologically negative, then irradiation of the axillary and supraclavicular basins in cN2b disease treats compartments in which disease has never been demonstrated and may represent overtreatment. This is a hypothesis rather than a recommendation, and the evidence base does not currently permit it to be resolved: The trials establishing a benefit from RNI delivered to the internal mammary component within comprehensive fields [47,48], and the DBCG-IMN cohort, which isolated the internal mammary component itself, did so against a background of periclavicular irradiation delivered in both groups [57]. No dataset therefore dissociates the contribution of the axillary and supraclavicular fields from that of the IMC. The uncertainty is also asymmetric, since occult axillary disease cannot be excluded while the FNR of TAD/SLNB remains incompletely validated in this population. A dedicated trial in cN2b disease could address both questions within a single design: patients with a pathologically negative axilla after TAD/SLNB randomised to IMC-restricted irradiation versus comprehensive RNI, stratified by MRI-assessed internal mammary response and by subtype, with locoregional recurrence as the primary endpoint and arm morbidity, cardiopulmonary dose, and quality of life as co-secondary endpoints. Until such a trial reports, comprehensive RNI remains the appropriate default.

## 10. Conclusions

Two response-defined hypotheses emerge from the evidence reviewed. Where nodal pCR is confirmed by TAD/SLNB with a fully assessed level I/II basin and the disease is cN2a or otherwise level I/II-predominant, omission of cALND with retention of RNI represents the better-supported de-escalation hypothesis, applying to both HER2-positive disease and TNBC; omission of cALND and RNI together is a substantially more speculative extrapolation from NSABP B-51/RTOG 1304 and should remain within prospective evaluation. In TNBC, the case for surgical de-escalation is further supported by subtype-specific outcome data after neoadjuvant chemo-immunotherapy in ypN0-selected patients [30], while B-51’s exploratory TNBC subgroup analysis does not provide a reliable basis for treatment selection in cN2/N3 disease. Where residual disease persists, cALND omission with comprehensive RNI to the undissected axilla is a comparable hypothesis, by analogy with AMAROS, pending Alliance A011202 and TAXIS. In cN2b, cN3a and cN3c disease, and for the internal mammary component of cN3b, the involved compartment is not sampled by level I/II surgery, and comprehensive RNI remains indicated regardless of response. In cN2b specifically, however, the axilla is clinically uninvolved: cALND is not required, and axillary staging should be by SLNB alone. IMC coverage is retained by default, while omission of an IMC boost after an MRI-assessed complete response, and more broadly whether comprehensive RNI could be omitted or irradiation restricted to the IMC in such responders, is an investigational hypothesis that future studies should evaluate [40]. These positions, together with their evidence strength, are summarised in a unified decision framework (Table 5).

There is no evidence from any prospective cN2/N3-specific trial that cALND itself improves survival, and the largest registry cohort confined entirely to cN2b–N3c disease found no adjusted survival advantage for ALND over SLNB among women receiving radiotherapy [27]. cALND (±RNI) therefore remains the conservative, guideline-supported default rather than a proven survival-improving standard, and the direction of the accumulating evidence is towards a smaller incremental role for dissection than has been assumed, at least where nodal pCR has been documented and regional irradiation is delivered. The reasonable question is no longer only why cALND might be omitted but what it adds once modern systemic therapy has cleared the axilla. The resulting uncertainty is substantial and provides a reasonable basis for prospective evaluation under clinical equipoise; this should be communicated honestly to patients. The two components of de-escalation should not, however, be treated as equivalent: omission of cALND after pathological restaging is the better supported of the two, whereas omission of cALND and RNI together remains substantially more speculative.

Outside a clinical trial, de-escalation is not yet standard care. What may nonetheless be considered on an individual basis, in carefully selected patients with cN2a or otherwise level I/II-predominant disease who achieve a complete radiological axillary response, is omission of cALND after pathological restaging by TAD, with retrieval of the marked node documented—or, where no marked node is available or it cannot be retrieved, by SLNB with at least three sentinel nodes recovered using dual-tracer mapping—with RNI generally retained; this requires multidisciplinary discussion and documented shared decision-making in which the evidentiary uncertainty is made explicit. Omission of cALND and RNI together should preferably remain within prospective evaluation rather than being adopted as individualised practice. Patients should be counselled that the approach remains investigational and is not supported by cN2/N3-specific randomised evidence. The research priorities are a dedicated, adequately powered cN2/N3 FNR study and the RNI-omission and residual-disease trials called for in this review, including, in cN2b disease, response-adapted internal mammary irradiation and a formal comparison of IMC-restricted irradiation with comprehensive RNI after a pathologically negative axilla.

Molecular restaging may eventually complement both approaches: clearance of circulating tumour DNA (ctDNA) after NST is an emerging, investigational marker of systemic remission, and whether it can support the omission of further axillary treatment after confirmed pCR deserves prospective evaluation alongside these trials [58].

## Figures and Tables

**Table 1:** Summary of landmark axillary de-escalation trials and their applicability to cN2/N3 disease (full trial-level detail in Table 2).

Trial	Population	cN2/N3 Included?	Applicability to cN2/N3
ACOSOG Z1071	cN+, post-NST SLNB	38/756 (5%), cN2 only; cN3 excluded	FNR 0% (0/14 with residual disease), but very wide 95% CI (0–23.2%); hypothesis-generating only
SENTINA	cN+, post-NST SLNB	Not separately analysed; cN3 excluded	Not applicable
SN-FNAC/GANEA2	cN+, post-NST SLNB	Few cN2 (2.5–6%); cN3 excluded	Not powered for subgroup conclusions
NSABP B-51/RTOG 1304	cN1 → ypN0, RNI vs. no RNI	Excluded (cN1 only)	Cannot be extrapolated directly
Alliance A011202	cN1 residual disease, ALND vs. axillary RT	Excluded (cN1 only)	Cannot be extrapolated directly
TAXIS	cN+ (upfront or post-NST)	Partial (cN2a/N3a only); cN2b/N3c excluded	Awaiting outcome data; does not cover full cN2/N3 spectrum

SLNB, sentinel lymph node biopsy; ALND, axillary lymph node dissection; RNI, regional nodal irradiation; TAS, tailored axillary surgery; FNR, false-negative rate; CI, confidence interval.

**Table 2:** Key studies and trials informing axillary management after NST, with cN2/N3 applicability.

Study/Trial	Design	cN2/N3 Included?	Key Finding Relevant to cN2/N3
ACOSOG Z1071 [4]	Prospective multicentre cohort	38/756 patients (cN2)	In the cN2 subset, SLN detection 89.5% (34/38); of 26 with ≥2 SLNs excised, 12 had nodal pCR and FNR was 0% in the remaining 14 (95% CI 0–23.2%)—hypothesis-generating only
SENTINA [5]	Prospective multicentre cohort	Not analysed separately	SLN detection/FNR not reported specifically for cN2/N3
SN-FNAC [6]	Prospective multicentre cohort	10/145 (6%)	Too few cN2 cases for subgroup conclusions
GANEA2 [7]	Prospective multicentre cohort	22/896 (2.5%)	Too few cN2 cases for subgroup conclusions
TAD registry [8]	Prospective multicentre	Included alongside cN1; cN2/N3 patients not reported or analysed separately	FNR 4.3% for TAD in patients converting cN+ to cN0
Alliance A011202 [9]	RCT, ALND vs. axillary RT	No—cT1–3N1 only	Testing whether axillary RT can replace ALND for residual disease in cN1; definitive oncological results pending; does not address cN2/N3
NSABP B-51/RTOG 1304 [10]	RCT, RNI vs. no RNI after ypN0	No—cT1–3N1 only	RNI omission safe in cN1→ypN0; explicitly excluded cN2/N3, so cannot be extrapolated directly
Jaen et al. 2026 (Ann Surg Oncol, NCDB) [13]	Retrospective national registry, cN2/N3	20,402 (100%)	36.9% overall ypN0; highest in HER2+ (70%); 60.8% still underwent ALND regardless of nodal response
TAXIS [14,15]	RCT, TAS ± ALND + RT	cN2a/N3a eligible; cN2b/N3c excluded	Only trial enrolling a portion of the cN2/N3 spectrum; results pending
Garcia-Tejedor et al. 2021 [16]	Retrospective, cN2 or ≥3 suspicious nodes on US	221 (100%)	Axillary pCR 67.9% (HER2+), 45.2% (TNBC), 16.8% (HR+/HER2-); subtype was the strongest predictor
Guo et al. 2024 (The Breast, CSBrS-012) [17]	Retrospective multicentre, cN2 HER2+/TNBC only	709 (100%)	Axillary pCR 78% when breast pCR achieved (HR-/HER2+ 83.8%, triple-positive [HR+/HER2+] 75%, TNBC 73.7%); RR of residual disease 12.4 if no breast pCR
Oh et al. 2025 (npj Breast Cancer) [18]	Retrospective single-institution, cN2–3	276 (100%)	Axillary pCR 49.3%; combined imaging + subtype nomogram AUC 0.90 for predicting axillary pCR
Noronha et al. 2020 (JCO Glob Oncol) [19]	Retrospective single-institution, all subtypes	262 (100%, cN2 only)	Axillary pCR 36.6% (cN2), 58.7% (cN1); overall pCR 22.6%; not HER2+/TNBC-restricted
Sun et al. 2024 (J Am Coll Surg) [20]	Retrospective single-institution, 2012–2018	521 (100%, cN2–3; 73.3% cN3)	Axillary pCR 34.2%; breast + axillary pCR 19.4%; axillary pCR independently associated with longer EFS (HR 0.22); TNBC worse EFS (HR 1.74); distant metastasis rate 29.4% despite response
Seto et al. 2026 (Ann Surg Oncol) [21]	Retrospective single-institution, LABC post-NST	Not cN2/N3-restricted	US sensitivity 71%/PPV 76% (highest); PET-CT specificity 73%/NPV 70% (highest); MRI lowest overall; concordance highest in cN3 (OR 6.2 vs. cN1); no modality accurate enough to replace surgical staging
Lee et al. 2026 [22]	Prospective single-centre trial; TAD then completion ALND, same operation	55 analysed (cN1 >3 nodes, cN2 or cN3; 53% cN2 or above)	TAD success 96%; clipped node identified 100%; axillary pCR 49%; FNR of TAD 0% (95% CI 0–13%, *n* = 27 informative) vs. 11.1% for SLNB alone and 18.5% for clipped-node excision alone
MARI protocol [23]	Prospective single-institution registry, PET-staged, ≥4 involved nodes on FDG-PET/CT	218 (100%, cN2–3)	ALND omitted (RT alone) if MARI-node pCR (47%, 103/218); axillary recurrence low in both groups at median 44 months (2.9%, 3/103, pCR/RT vs. 3.5%, 4/115, non-pCR/ALND + RT); 5-yr iDFS 89% (95% CI 83–96) and OS 95% (95% CI 91–99) in the ypN0 group; 39% had extra-axillary (periclavicular/parasternal) nodal disease on FDG-PET/CT
Rangan et al. 2026 (Surgery) [24]	Retrospective single-institution cohort, NAC-treated, 2016–2024	217 (100%, cN2–3; 142 cN2, 75 cN3)	Nodal pCR 46.5% (cN2) and 52.0% (cN3); 4 axillary recurrences (1.8%) at median 46 months, all after upfront ALND, none among the 95 patients undergoing SLN surgery; 5-yr OS 75.9% (SLN) vs. 87.5% (SLN + ALND) vs. 85.3% (ALND), *p* = 0.056; 5-yr RFS 88.8% vs. 73.9% vs. 69.7%, *p* = 0.022; complete axillary imaging response predicted nodal pCR in 43/44 (97.7%); no FNR data, as no completion ALND was performed in the SLN-alone group; 92.6% received adjuvant radiotherapy
Shin et al. 2025 (Front Oncol) [25]	Retrospective single-institution cohort of sentinel-node-negative patients, 2008–2021	432 (100%, cN2–3; 148 cN2, 284 cN3)	Axillary recurrence 2.3% (10/432) at median 44 months; 5-yr ARFS 97.3% with SLNB alone (*n* = 297) vs. 92.9% with SLNB + cALND (*n* = 135), *p* = 0.415; RRFS and OS likewise not different, before or after propensity-score matching, including where internal mammary (*n* = 180) or supraclavicular (*n* = 98) nodes were involved; however, 6/135 (4.4%) undergoing completion cALND after a negative sentinel node had further nodal metastases, 5 of them with ≥4 involved nodes
Park TS et al. 2018 (Ann Surg Oncol, NCDB) [26]	Retrospective national registry, 2004–2013	22,156 (100%)	ALND associated with improved OS vs. SLNB (HR 0.68); not NST-response-stratified, confounding by indication likely
Roach et al. 2024 (Ann Surg Oncol, NCDB) [27]	Retrospective national registry, adjuvant RT-treated, 2012–2017	7167 (100%, cN2b–N3c)	87% received ALND vs. 13% SLNB; after IPTW adjustment, ALND not associated with improved OS vs. SLNB (HR 0.99, *p* = 0.91)—contrasts with the unadjusted signal in [26]
Piltin et al. 2020 (Ann Surg Oncol, Mayo Clinic) [28]	Retrospective single-institution, cN1–3, NST-treated	Included alongside cN1 (cN1–3); cN2/N3 proportion not reported, and recurrence not stratified by initial cN category	2-yr regional recurrence-free rate 99.1% (SLN surgery) vs. 96.4% (ALND), *p* = 0.10; 1 regional recurrence among 159 patients treated with SLN surgery alone, not stratified by initial cN category; selection for SLN surgery significantly favoured cN1; indirect evidence only; short follow-up
NEOSENTITURK MF-1803 [29]	Prospective multicentre registry, 37 centres	Included alongside cN1 (cN1–3): 792 cN1, 153 cN2, 31 cN3	184/976 (18.9%) cN2–3 (153 cN2, 31 cN3); 3 axillary recurrences in total (1/356 TAD vs. 2/620 SLNB, 0.3% vs. 0.3%) reported by surgical procedure, not by initial cN stage, so no cN2–3 numerator is available; all patients received RNI including the axilla; cN2–3 independently associated with lower 5-yr disease-specific survival vs. cN1 (96.8% vs. 98.7%, *p* = 0.03); cN2–3 axillary-specific FNR not separately reported; supportive, indirect evidence
Li et al. 2026 (Front Immunol, NCDB) [30]	Retrospective national registry, TNBC, NAC + immunotherapy-treated	4336 (cN1–3), of whom 678 cN2/N3	In ypN0-selected patients, SLNB vs. ALND 5-yr OS comparable (90.1% vs. 93.6%, *p* = 0.99); no survival detriment on multivariable analysis (aHR 1.19, 95% CI 0.54–2.61); selection required confirmed nodal pCR, not unconfirmed clinical/radiological response
Qian et al. 2022 (Front Oncol) [31]	Retrospective single-institution, 2010–2021	292 pCR/near-pCR patients (subset cN3)	cN3 independently predicted recurrence after both pCR (*p* = 0.003) and near-pCR (*p* = 0.036); 5-yr RFS 94.6% (pCR) vs. 85.6% (near-pCR)
Park HJ et al. 2011 (Int J Radiat Oncol Biol Phys) [32]	Retrospective single-institution, PET-staged	55 (100%, cN3)	Only 5.5% failed at original PET-positive nodal site; RT to PET-positive IMN (cN3b) or dose escalation ≥55 Gy to SCL (cN3c) not associated with improved 5-yr IMN/SCL RFS or DFS; dominant failure was distant, not regional

US, ultrasound; FNR, false-negative rate; RT, radiotherapy; TAS, tailored axillary surgery; TAD, targeted axillary dissection; RNI, regional nodal irradiation; SLN, sentinel lymph node; ALND, axillary lymph node dissection; HR, hazard ratio (hormone receptor where written HR+/HR−); aHR, adjusted hazard ratio; RR, relative risk; CI, confidence interval; AUC, area under the curve; PPV, positive predictive value; NPV, negative predictive value; EFS, event-free survival; OS, overall survival; NCDB, National Cancer Database; IPTW, inverse probability of treatment weighting. All trials explicitly excluded cN3 disease unless stated otherwise; the principal exception is TAXIS, which permits cN2a and cN3a disease while excluding cN2b and cN3c (see the cN2/N3-inclusion column for each trial’s specific inclusions). Studies listed as including cN2/N3 patients “alongside cN1” are mixed cN1–3 cohorts in which cN2/N3 patients form a minority; unless a cN2/N3-specific numerator and denominator are given in the final column, their results cannot be attributed to the cN2/N3 subgroup and should be read as indirect evidence.

**Table 3:** Breast and axillary pCR rates by subtype in 709 cN2 patients after NST, restricted to HER2-positive disease—comprising both the triple-positive (HR-positive/HER2-positive) and the HR-negative/HER2-positive variants—and TNBC; HR-positive/HER2-negative disease was excluded. Data from Guo et al., The Breast 2024 [17].

Subtype	Breast pCR Rate	Axillary pCR Rate (Overall)	Axillary pCR Given Breast pCR
HR-/HER2+	32.2%	42.2%	83.8%
Triple-positive (HR+/HER2+)	19.3%	33.8%	75.0%
Triple-negative	24.9%	34.1%	73.7%
All cN2 (*n* = 709)	25.0%	36.4%	78.0%

HR, hormone receptor; HER2, human epidermal growth factor receptor 2; TNBC, triple-negative breast cancer; pCR, pathological complete response.

**Table 4:** Summary of key studies evaluating real-world oncological outcomes following axillary de-escalation in cN2/N3 breast cancer.

Study & Design	Patient Population	N	Key Oncological Outcomes	Key Limitations & Clinical Notes
MARI-protocol registry [23], prospective (2014–2021)	cN2–3-equivalent disease (≥4 involved nodes on FDG-PET/CT); 39% with extra-axillary nodal disease	218	Axillary recurrence: 2.9% (MARI-pCR + RT) vs. 3.5% (non-pCR + ALND + RT) at 44 months. Five-year iDFS 89% (95% CI 83–96) and OS 95% (95% CI 91–99) in the ypN0 group treated with RT alone.	Non-randomised, single-arm, single-institution registry; response-based selection onto the RT-alone pathway; 44-month median follow-up; mixed receptor subtypes without subtype-specific outcomes; 39% had extra-axillary nodal disease on FDG-PET/CT, which level I/II surgery does not sample.
Rangan et al. 2026 [24], retrospective single-institution cohort (2016–2024)	cN2 (*n* = 142) and cN3 (*n* = 75) disease, all NAC-treated	217	Axillary recurrence in 4 patients (1.8%), all after upfront ALND; 0 of 60 treated with SLN surgery alone and 0 of 35 treated with SLN surgery plus ALND. These sat within 9 locoregional (4.2%) and 30 distant (13.8%) recurrences overall; crude recurrence of any type was 6.7% (SLN), 14.3% (SLN + ALND) and 24.6% (ALND), *p* = 0.010. Five-year OS 75.9% (SLN), 87.5% (SLN + ALND), 85.3% (ALND), *p* = 0.056; five-year RFS 88.8%, 73.9%, 69.7%, *p* = 0.022. Complete axillary imaging response predicted nodal pCR in 43 of 44 patients (97.7%).	Restricted entirely to cN2/N3 disease, with an extractable axillary-recurrence numerator. Retrospective; selection was overt (extranodal extension, lymphovascular invasion, imaging response and residual nodal burden all differed between groups), so between-group comparisons reflect prognosis as much as extent of surgery; no completion ALND in the SLN-alone group, so no FNR estimate is possible; 92.6% received adjuvant radiotherapy.
Shin et al. 2025 [25], retrospective single-institution cohort (2008–2021)	cN2 (*n* = 148) and cN3 (*n* = 284) disease with a negative sentinel node biopsy after NAC	432	Overall axillary recurrence 2.3% (10/432) at median 44 months. Five-year ARFS 97.3% (SLNB alone) vs. 92.9% (SLNB + cALND), *p* = 0.415; RRFS 96.0% vs. 95.8%, *p* = 0.689; OS 94.2% vs. 87.6%, *p* = 0.972. After propensity-score matching (124 per group), five-year ARFS 99.2% vs. 96.6%, *p* = 0.823. No difference by axillary strategy in the subgroups with internal mammary (*n* = 180) or supraclavicular (*n* = 98) involvement.	Restricted entirely to cN2/N3 disease and the only cohort here reporting outcomes by extra-axillary involvement. Retrospective, single-centre, with surgeon-determined allocation; 2008–2021 era predates contemporary regimens; 95.6% received radiotherapy, so axillary control reflects a combined strategy. Sentinel-node-positive patients were excluded by design, so no FNR can be derived; note that 6 of the 135 patients undergoing completion cALND (4.4%) harboured further nodal metastases despite a negative sentinel node, 5 of them with ≥4 involved nodes.
Park TS et al. 2018 [26], retrospective NCDB analysis	cN2–3 disease (2004–2013)	22,156	ALND was associated with improved OS compared with SLNB after adjustment (HR 0.68, *p* < 0.001).	Predominantly upfront-surgery era; only 39.7% received neoadjuvant systemic therapy; high risk of confounding by indication.
Roach et al. 2024 [27], retrospective NCDB analysis	cN2b–N3c disease treated with adjuvant RT	7167	After IPTW adjustment, ALND showed no survival advantage over SLNB (HR 0.99, *p* = 0.91).	Retrospective database design; treatment-selection bias; findings contrast with [26]. Neoadjuvant exposure and ypN status not characterised; overall survival the only endpoint, with no axillary-recurrence data.
Selfridge et al. 2025 [41], retrospective NCDB analysis	cN3b disease (axillary plus ipsilateral internal mammary nodes), 2012–2021	4236	SLNB alone rose from 8.5% (2012) to 35.1% (2021); nodal pCR 24.9% after NAC; among patients with nodal pCR, OS after SLNB or SLNB + ALND did not differ from ALND alone (*p* = 0.22 and 0.57).	Retrospective database design; selection bias; no locoregional-recurrence endpoint; radiotherapy fields not accounted for; applies only to pathologically confirmed nodal pCR.
Piltin et al. 2020 [28], retrospective Mayo Clinic cohort	cN1–3 disease	602	Two-year freedom from regional recurrence: 99.1% with SLNB vs. 96.4% with ALND (*p* = 0.10). One regional recurrence among the 159 patients treated with SLN surgery alone.	Indirect evidence. Recurrence not stratified by initial cN category, so no cN2/N3-specific estimate is extractable; selection for SLN surgery was significantly associated with lower presenting nodal category (cN1 rather than cN2–3); a subsequent review describes the 159 SLN-surgery-alone patients as pre-neoadjuvant cN1, which requires reconciliation against the primary report. Short follow-up.
NEOSENTITURK MF-1803 [29], prospective registry	cN1–3 disease across 37 centres (792 cN1, 153 cN2, 31 cN3)	976	Axillary recurrence: 3 events in total, 1 of 356 with TAD vs. 2 of 620 with SLNB (0.3% vs. 0.3%), reported by surgical procedure rather than by initial cN stage. Five-year DSS: cN2–3 96.8% vs. cN1 98.7% (*p* = 0.03).	Indirect evidence. Predominantly cN1 (792 of 976, 81.1%); only 184 (18.9%) cN2–3, with no axillary-recurrence numerator reported for them, so they cannot enter a pooled high-burden analysis. All patients received RNI including the axilla. cN2–3 subgroup not separately evaluated for FNR; pretreatment nodal burden remained prognostic despite treatment response.
Li et al. 2026 [30], retrospective TNBC NCDB analysis	cN1–3 TNBC receiving neoadjuvant chemo-IO; cN2/3 subgroup with confirmed nodal pCR	4336 total; 678 cN2/3	Five-year OS: 90.1% with SLNB vs. 93.6% with ALND (*p* = 0.99). Multivariable analysis: aHR 1.19, *p* = 0.67.	Restricted to patients with confirmed nodal pCR (ypN0); does not address FNR when nodal response is unconfirmed.

Abbreviations: ALND, axillary lymph node dissection; aHR, adjusted hazard ratio; ARFS, axillary recurrence-free survival; cN, clinical nodal stage; DSS, disease-specific survival; FNR, false-negative rate; HR, hazard ratio; iDFS, invasive disease-free survival; IO, immunotherapy; IPTW, inverse probability of treatment weighting; MARI, marking the axilla with radioactive iodine seeds; NCDB, National Cancer Database; OS, overall survival; pCR, pathological complete response; RFS, recurrence-free survival; RRFS, regional recurrence-free survival; RT, radiotherapy; SLNB, sentinel lymph node biopsy; TAD, targeted axillary dissection; TNBC, triple-negative breast cancer; ypN0, pathological node-negative status after neoadjuvant systemic therapy. Only the MARI-protocol registry, Rangan et al. and Shin et al. are restricted entirely to advanced nodal disease and able to supply an axillary-recurrence numerator and denominator. Park TS et al., Roach et al. and Selfridge et al. are likewise confined to cN2/N3 disease but report survival alone, with no locoregional endpoint. The cohorts listed as cN1–3 include cN1 patients alongside cN2/N3 and do not report axillary-recurrence events separately for their cN2/N3 subgroups. Both of the latter groups are therefore supportive rather than directly applicable.

**Table 5:** A unified decision framework for cN2/N3 axillary management, aligning the positions developed earlier in this review.

Clinical Situation	Evidence Strength	Suggested Position
cN2/N3, no pathological nodal assessment (imaging/clinical response only)	Very weak (no cN2/N3 prospective validation)	Do not de-escalate axillary surgery or RNI on imaging/clinical response alone; pathological nodal assessment remains required
cN2/N3, TAD/SLNB-confirmed nodal pCR, HER2-positive, cN2a or level I/II-predominant disease	Retrospective and extrapolated (B-51/RTOG 1304 is cN1-derived)	RNI omission is an investigational hypothesis warranting prospective evaluation, not routine practice
cN2/N3, TAD/SLNB-confirmed nodal pCR, TNBC	Retrospective and extrapolated—B-51’s TNBC subgroup was an exploratory analysis with few events (RNI associated with numerically worse outcomes, HR 2.30 for RNI, 95% CI 1.00–5.25, lower bound approaching 1); cN2/N3 was excluded from B-51	RNI omission is a hypothesis warranting prospective evaluation; the exploratory TNBC subgroup finding should not itself be used as a positive rationale for omission—pursue only via MDT discussion and shared decision-making, as for HER2-positive disease
cN2/N3, residual nodal disease after NST	No cN2/N3 RCT (Alliance A011202 and TAXIS are cN1-only or partial)	cALND (± RNI) remains the conservative default pending trial results
cN2b (internal mammary involvement without axillary involvement)	No cN2/N3-specific data; the IMC is not assessable by level I/II surgery, and nodal irradiation trials delivered IMC coverage within comprehensive fields	cALND is not required; axillary staging by SLNB alone is appropriate. IMC coverage is retained by default. Omission of an IMC boost after an MRI-assessed complete response, and restriction of irradiation to the IMC with omission of the axillary and supraclavicular fields, are hypotheses for prospective trials, not current practice
cN3a (infraclavicular) or cN3c (supraclavicular) disease specifically	Anatomically distinct—level I/II TAD/SLNB does not sample these compartments	Comprehensive RNI to the involved compartment remains indicated regardless of level I/II nodal pCR status
More than three suspicious but mobile, non-matted nodes (AJCC cN1 by fixation criteria; count-defined high nodal burden)	Count-defined rather than AJCC-defined; this group comprised approximately half of the only prospective TAD accuracy cohort in high nodal burden	Disease lies wholly within the level I/II basin, so the anatomical argument applies in full; manage as for cN2a or otherwise level I/II-predominant disease, with TAD and documented marked-node retrieval preferred

cALND, completion axillary lymph node dissection; RNI, regional nodal irradiation; TAD, targeted axillary dissection; SLNB, sentinel lymph node biopsy; RCT, randomised controlled trial; NST, neoadjuvant systemic therapy; IMC, internal mammary chain. This framework does not constitute a validated treatment algorithm; every position outside the first row remains an unvalidated, hypothesis-generating extrapolation pending prospective, cN2/N3-specific trials.

## Data Availability

Not applicable—this is a narrative review, and no new data were generated or analysed.

## Supplementary Materials

The following supporting information can be downloaded at https://www.mdpi.com/article/10.3390/cancers18182986/s1. Table S1: SANRA (Scale for the Assessment of Narrative Review Articles) quality self-assessment of this review, with the manuscript location and justification for each of the six items.

## Abbreviations

AJCC, American Joint Committee on Cancer; ALND, axillary lymph node dissection; AUC, area under the curve; cALND, completion axillary lymph node dissection; CI, confidence interval; EFS, event-free survival; FNR, false-negative rate; HER2, human epidermal growth factor receptor 2; HR, hazard ratio or hormone receptor; IMC, internal mammary chain; IPTW, inverse probability of treatment weighting; LABC, locally advanced breast cancer; MDT, multidisciplinary team; MLNB, marked lymph node biopsy; NAC, neoadjuvant chemotherapy; NCDB, National Cancer Database; NST, neoadjuvant systemic therapy; NPV, negative predictive value; OS, overall survival; pCR, pathological complete response; PPV, positive predictive value; RNI, regional nodal irradiation; RR, relative risk; SLN(B), sentinel lymph node (biopsy); SPIO, superparamagnetic iron oxide; TAD, targeted axillary dissection; TAS, tailored axillary surgery; TNBC, triple-negative breast cancer; VAB, vacuum-assisted biopsy; ycN0, clinically node-negative after neoadjuvant therapy; ypN0, pathologically node-negative after neoadjuvant therapy.
