# Supplementary material for: Axillary De-Escalation After Neoadjuvant Therapy in cN2/N3 HER2-Positive and Triple-Negative Breast Cancer: Navigating Biological Promise and Clinical Uncertainty
Source: Cancers (Basel). 2026 Sep 15;18(18):2986. doi: 10.3390/cancers18182986 (PMC13604889; doi:10.3390/cancers18182986)
Supplement: Supplementary file 1 [file cancers-18-02986-s001.zip › cancers-4556466-supplementary.pdf]

Supplementary Material

SANRA — Scale for the Assessment of Narrative Review Articles: Author Self-Assessment

Manuscript: Axillary De-Escalation After Neoadjuvant Therapy in cN2/N3 HER2-Positive and Triple-Negative Breast Cancer: Navigating Biological Promise and Clinical Uncertainty

Article type: Review

Each SANRA item is scored 0 (low standard), 1 (moderate standard) or 2 (high standard); maximum total score 12.

SANRA item	Max	Score	Location and justification in the manuscript
1. Justification of the article's importance for the readership	2	2	Introduction (Section 1) and Abstract (Background and Objective): the review explains that landmark axillary de-escalation trials largely excluded cN2/N3 disease while contemporary NST produces high nodal pCR rates in HER2-positive and triple-negative breast cancer, creating a clinically important evidence gap for readers managing these patients.
2. Statement of concrete aims or formulation of questions	2	2	Abstract (Background and Objective) and end of Introduction (Section 1): the review states explicit questions — whether high nodal pCR rates could support axillary de-escalation (TAD/SLNB instead of cALND; RNI omission or substitution) in cN2/N3 disease, and which evidence gaps prevent adoption. Sections 4, 6 and 7 are framed as specific questions.
3. Description of the literature search	2	2	Methods (Search Strategy) (Section 2): databases (PubMed/MEDLINE, Google Scholar, ClinicalTrials.gov), search terms, date of last search (31 August 2026), language restriction, records identified/retained (453/54), hand-searching of reference lists and consensus statements, and explicit study-selection and prioritisation criteria are reported. The narrative (non-PRISMA) nature of the search is stated explicitly.
4. Referencing	2	2	Throughout the manuscript: key statements on pCR rates, FNR, trial eligibility, oncological outcomes and guideline positions are supported by numbered references to 54 sources (Vancouver style, numerals in round brackets), favouring landmark trials, prospective registries and large national database analyses; conference abstracts are identified as such in the reference list.
5. Scientific reasoning	2	2	Sections 3–7, 9 and 10: the argument distinguishes biological plausibility (nodal pCR rates) from technical eligibility (unvalidated TAD/SLNB false-negative rate in cN2/N3) and trial-validated eligibility; evidence levels are labelled (prospective vs retrospective, exploratory subgroup analyses), and extrapolations from cN1-derived trials (B-51, A011202, AMAROS) are explicitly identified as hypothesis-generating. AJCC nodal subcategories are separated out rather than treated as one entity: cN2b is handled distinctly (cALND not required, IMC coverage retained), and the proposition that axillary and supraclavicular irradiation may constitute overtreatment in that setting is framed as a hypothesis for a specific future trial rather than a practice position, with the reasons it cannot be resolved from existing data stated. Limitations are addressed in Section 9.
6. Appropriate presentation of data	2	2	Tables 1–5 and Figures 1–3: endpoint data are presented with effect sizes and precision where available (e.g., FNR with 95% CIs; HR 2.30, 95% CI 1.00–5.25 for the exploratory B-51 TNBC subgroup; survival percentages with P values), and a unified decision framework (Table 5, including a dedicated cN2b row) and conceptual pathway (Figure 1) summarise the evidence-to-hypothesis mapping.
**Total**	**12**	**12**	

Reference: Baethge C, Goldbeck-Wood S, Mertens S. SANRA — a scale for the quality assessment of narrative review articles. Res Integr Peer Rev. 2019;4:5. doi:10.1186/s41073-019-0064-8.
